# Supplementary material for: Grid-like entorhinal representation of an abstract value space during prospective decision making
Source: Nat Commun. 2024 Feb 9;15:1198. doi: 10.1038/s41467-024-45127-z (PMC10858181; doi:10.1038/s41467-024-45127-z)
Supplement: Supplementary file 1 — Supplementary Information [file 41467_2024_45127_MOESM1_ESM.pdf]

Grid-like entorhinal representation of an abstract value space during prospective decision making

Alexander Nitsch[1], Mona M. Garvert[1,2,3,4], Jacob L. S. Bellmund[1], Nicolas W. Schuck[2,3,5], Christian F. Doeller[1,6,7,8]

[1] Max Planck Institute for Human Cognitive and Brain Sciences, Leipzig, Germany
[2] Max Planck Research Group NeuroCode, Max Planck Institute for Human Development, Berlin, Germany
[3] Max Planck UCL Centre for Computational Psychiatry and Aging Research, Berlin, Germany
[4] Faculty of Human Sciences, Julius-Maximilians-Universität Würzburg, Würzburg, Germany
[5] Institute of Psychology, Universität Hamburg, Hamburg, Germany
[6] Kavli Institute for Systems Neuroscience, Centre for Neural Computation, The Egil and Pauline Braathen and Fred Kavli Centre for Cortical Microcircuits, Jebsen Centre for Alzheimer's Disease, Norwegian University of Science and Technology, Trondheim, Norway
[7] Wilhelm Wundt Institute for Psychology, Leipzig University, Leipzig, Germany
[8] Department of Psychology, Technical University Dresden, Dresden, Germany
Correspondence: nitsch@cbs.mpg.de or doeller@cbs.mpg.de

Supplementary Information

Supplementary Fig. 1	Task design: Trajectories through the value space
Supplementary Fig. 2	Supplementary behavioral analyses of the prospective decision making task
Supplementary Fig. 3	Correlation of the prospective decision making task with the two-stage task
Supplementary Fig. 4	Supplementary analyses of the entorhinal grid-like value representation
Supplementary Fig. 5	Prospective value difference effects after controlling for reaction time
Supplementary Fig. 6	Prospective value difference effects when including only correct trials
Supplementary Fig. 7	Prospective value difference effects after controlling for the distance between the choice location and the 45°-diagonal
Supplementary Fig. 8	Occipital-temporal representations of choice options
Supplementary Fig. 9	Occipital-temporal representations of choice options when using only switch time points
Supplementary Fig. 10	Occipital-temporal representations of choice options when using only incorrect trials

Supplementary Table 1	Exploratory whole-brain clusters of the hexadirectional modulation effect
Supplementary Table 2	Significant clusters of the value difference effect
Supplementary Table 3	Significant clusters of the prospective value difference effect

Supplementary Figures

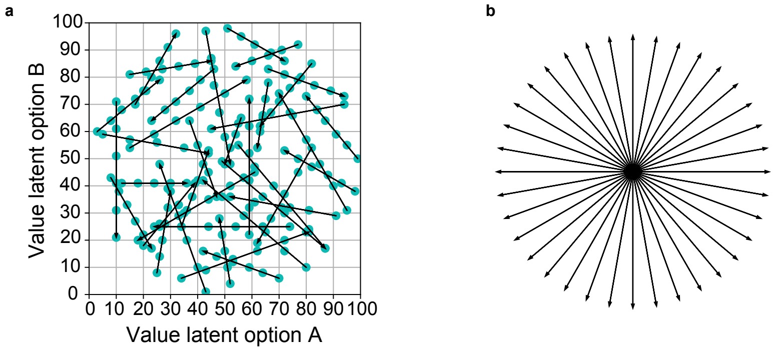

Supplementary Fig. 1 | Task design: Trajectories through the value space. a Example of trajectories through the value space in a task block. Arrows depict trajectories, and dots along trajectories depict the time points. **b** In each task block, directions of trajectories were sampled homogeneously from 0°-350° in 10°-steps.

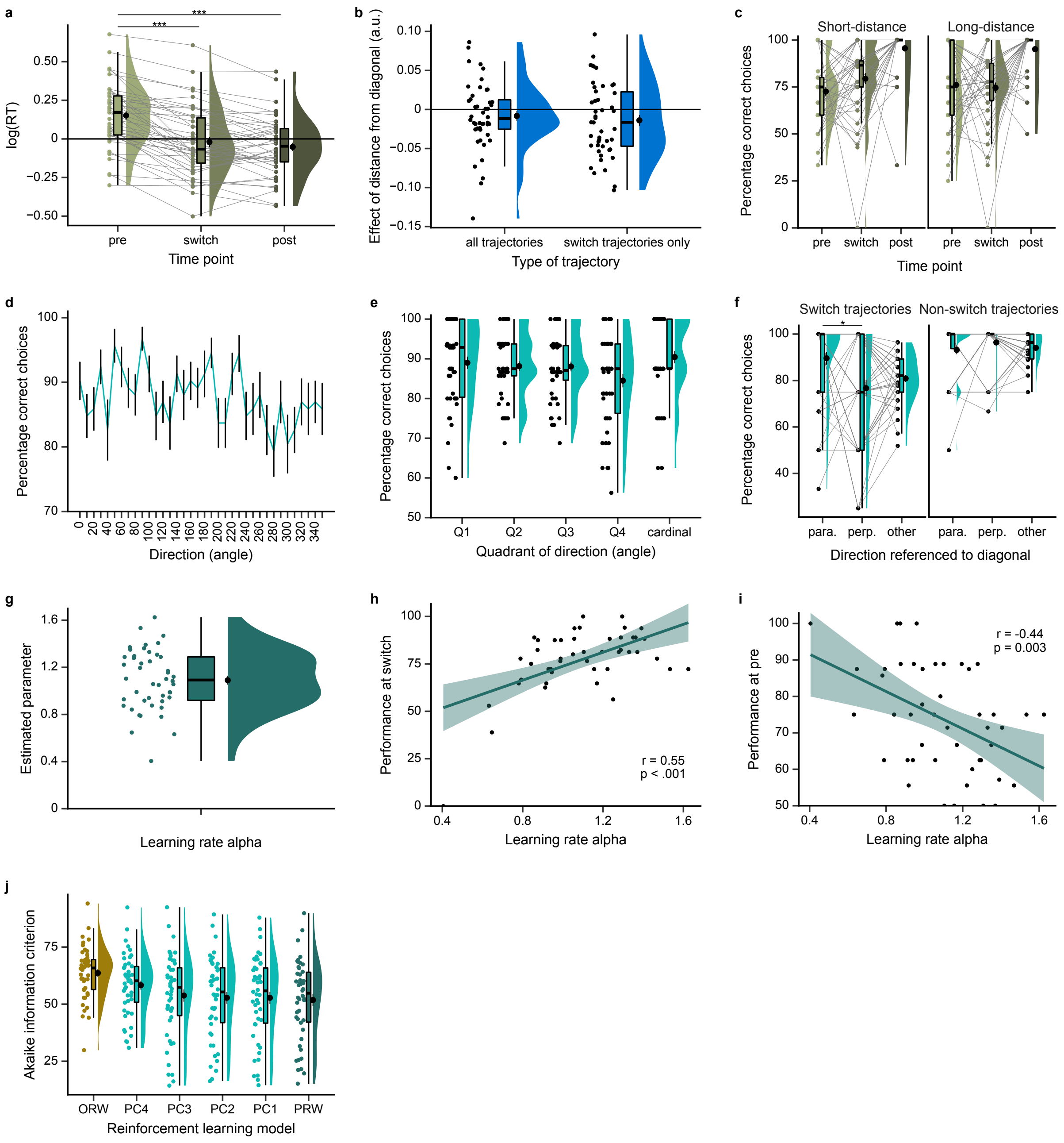

Supplementary Fig. 2 | Supplementary behavioral analyses of the prospective decision making task. a Reaction times (log) along different time points in switch trajectories, at the time point before the switch (pre), of the switch (switch) and after the switch (post). There was a significant effect of time point on reaction times (repeated measures ANOVA with Bonferroni-corrected post-hoc related-samples *t*-tests (α = 0.016): $F(2,90)$ = 60.65, p < .001; post hoc pairwise tests: pre-post: $t(45)$ = 9.85, p < .001, switch-post: $t(45)$ = 1.74, p = .09; pre-switch: $t(45)$ = 8.33, p < .001). **b** Effect of the distance between the choice location and the 45°-diagonal of the value space on reaction times, separately for all trajectories (left) and only switch trajectories as a control (right). In both cases, effect sizes estimated by participant-specific linear regressions were not significantly different from 0 (one-sample *t*-tests; all trajectories: $t(45)$ = -1.29, p = .20; switch trajectories: $t(45)$ = -2.01, p = .051). **c** Performance along different time points in switch trajectories (pre, switch, post), separately for the two types of trajectories with regard to the distance between two consecutive time points (see Methods): trajectories with a relatively smaller distance between time points (short-distance trajectories) or a relatively larger distance (long-distance trajectories). We reasoned that variation in the distance between time points would place different demands on extrapolating values. Previous piloting work indicated performance differences between these two distance levels, with higher performance at the switch and lower performance at the pre time point in short-distance compared to the long-distance trajectories. Contrary to our expectations based on previous piloting, we did not observe a significant interaction between distance type and time points (repeated measures ANOVA; interaction effect: $F(2,90)$ = 1.40, p = .25; main effect of distance type: $F(1,45)$ = 0.16, p = .69; direct comparison of the switch time point with one-sample *t*-test: $t(45)$ = 1.69, p = .097). **d** Performance for different directions (angles) of

trajectories. Line depicts mean performance and error bars correspond to the standard error of the mean. **e** Performance for different quadrants of directions (angles). Q1 refers to directions 10°-80°, Q2 to 100°-170°, Q3 to 190°-260°, Q4 to 280°-350° and cardinal refers to directions 0°, 90°, 180° and 270°. We observed an effect of quadrant, with higher performance for cardinal vs. Q4 directions (repeated measures ANOVA with Bonferroni-corrected post-hoc related-samples *t*-tests ($\alpha = 0.005$): $F(4,180) = 2.93$, $p = .02$; post hoc pairwise tests: cardinal-Q4: $t(45) = 3.01$, $p = .004$; all other comparisons n.s. $p > .005$). **f** Performance for trajectories with directions approximately parallel to the 45°-diagonal (sampled directions: 40°, 50°, 220°, 230°), approximately perpendicular to the 45°-diagonal (sampled directions: 130°, 140°, 310°, 320°) and all other directions, separately for switch and non-switch trajectories. There was a significant interaction between switch vs. non-switch trajectories and direction (repeated measures ANOVA; interaction: $F(2,86) = 7.18$, $p = .001$; main effect direction: $F(2,86) = 3.20$, $p = .045$; main effect switch: $F(1,43) = 59.06$, $p < .001$). We further investigated the interaction effect using post-hoc related-samples *t*-tests with Bonferroni correction ($\alpha = .008$, Bonferroni-corrected for 6 pairwise tests). This revealed significantly higher performance for parallel vs. perpendicular directions in switch trajectories only ($t(43) = 2.90$, $p = .005$; comparison parallel vs. other in switch trajectories: $t(43) = 2.67$, $p = .014$ n.s. after correction; all other pairwise comparisons n.s. $p > .008$). **g** Estimated parameters of the learning rate α of the prospective Rescorla-Wagner model. A learning rate of 1 reflects full updating of values according to prediction errors and value changes, learning rates above 1 hence suggest slight over-updating. **h** Significant positive correlation between the learning rate α of the prospective Rescorla-Wagner model and performance at the switch time point (Pearson $r(44) = .55$, $p < .001$; after exclusion of outlier with performance=0: $r(44) = .41$, $p = .005$). **i** Significant negative correlation between the learning rate α of the prospective Rescorla-Wagner model and performance at the pre time point (Pearson $r(44) = -.44$, $p = .003$). **j** Reinforcement learning model comparison for all models including alternative control models. ORW refers to the original Rescorla-Wagner (RW) model and PRW refers to the prospective Rescorla-Wagner (RW) model as depicted in Fig. 2 of the main text. PC refers to prospective control models and the numbers correspond to numbers of these control models in the Methods section. Depicted is the Akaike information criterion (AIC), with lower values indicating better model fit. Models are ordered according to their mean AIC from left to right. The prospective Rescorla-Wagner model (PRW) fitted the data better than any control model (related-samples *t*-tests with Bonferroni correction, all $p < .001$, see Methods for details). **a-c,e-g,j** Dots represent data from $n = 46$ participants; boxplots show median and upper/lower quartile with whiskers extending to the most extreme data point within 1.5 interquartile ranges above/below the quartiles; black circles with error bars correspond to mean ± SEM; distributions depict probability density functions of data points. **h,i** Dots represent data from $n = 46$ participants; line represents linear regression line, with shaded regions as the 95% confidence interval. * $p < .05$ corr., *** $p < .001$. All statistical tests were two-sided. Source data are provided as a Source Data file.

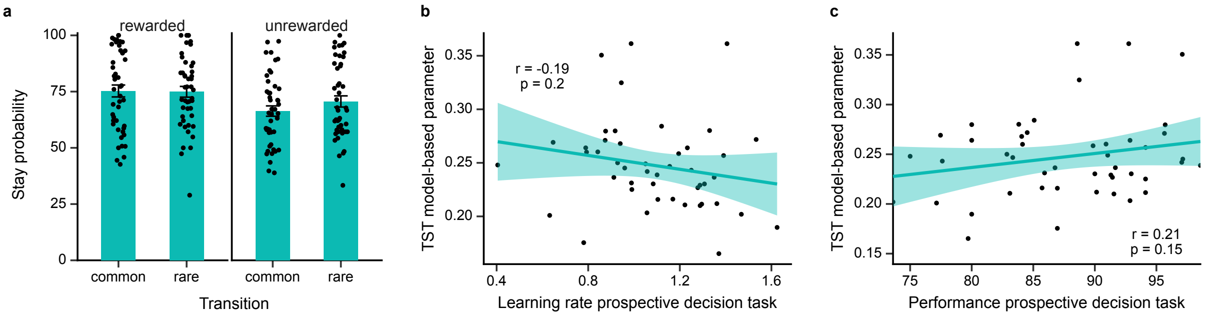

Supplementary Fig. 3 | Correlation of the prospective decision making task with the two-stage task. a Two-stage task: Probability of repeating a first-stage choice (staying) as a function of reward and transition type in the preceding trial. As expected, we observed a significant effect of reward (repeated measures ANOVA; $F(1,45) = 20.50$, $p < .001$) and a significant interaction of reward and transition type ($F(1,45) = 4.38$, $p = .04$; main effect transition type: $F(1,45) = 3.37$, $p = .07$). In addition, we fitted choice behavior with the hybrid reinforcement learning model as described in Daw et al. (2011)[1] to extract a model-based parameter estimate per participant (y-axis in **b-c,** parameter ranging from 0=model-free to 1=model-based). To our surprise, model-based behavior in our sample was less pronounced than typically observed, given the relatively smaller interaction effect of reward and transition type as well as lower estimates of the model-based parameter compared to Daw et al. (2011)[1]. We speculate that this might be partly attributed to participants' exhaustion after the MRI session when performing the task at the end of the study. **b-c** We did not observe any significant correlations between the model-based parameter and the learning rate ($p = .20$) or performance ($p = .15$) in our prospective decision making task (Pearson correlation with Bonferroni correction for two tests). We speculate that the restricted range of the model-based parameter might have contributed to the lack of a correlation. **a-c** Dots represent data from $n = 46$ participants; barplots showing mean ± SEM; lines represent linear regression lines, with shaded regions as the 95% confidence intervals. All statistical tests were two-sided. Source data are provided as a Source Data file.

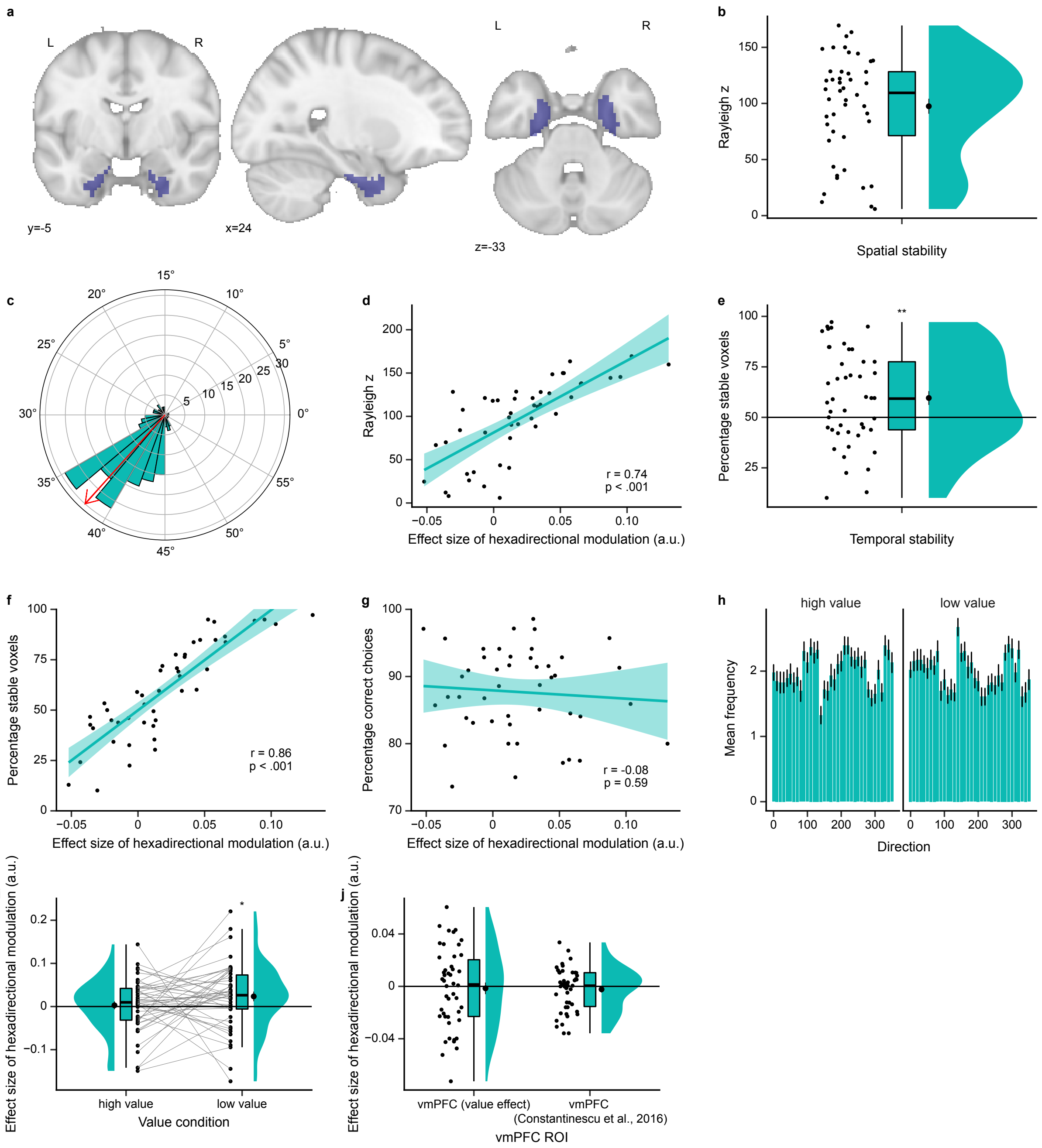

Supplementary Fig. 4 | Supplementary analyses of the entorhinal grid-like value representation. a Entorhinal cortex mask used for small volume correction on the group level. Mask is displayed on the MNI template. **b-f** Control analyses to examine the stability of grid orientations in the significant entorhinal cluster. The magnitude of the hexadirectional modulation should depend on both spatial and temporal stability of grid orientations. **b** Regarding spatial stability, we observed significant clustering of orientations across voxels within-participant. Depicted are the Rayleigh z statistics of the Rayleigh test for non-uniformity of circular data. The test indicated significant deviation from uniformity of voxel orientations in all participants ($p < .05$ in all participants). **c** To illustrate this spatial stability, this plot shows a polar histogram of voxel orientations in the significant entorhinal cluster of an example participant (median Rayleigh z statistic). Red arrow denotes the mean orientation. **d** Across participants, this spatial stability correlated significantly positively with the magnitude of the hexadirectional modulation (Pearson $r(44) = .74$, $p < .001$). The effects in **b** and **d** were still present when analyzing spatial stability in unsmoothed data (Rayleigh test for non-uniformity of circular data: $p < .05$ in 41 of 46 participants; correlation: $r(44) = .85$, $p < .001$). **e** Regarding temporal stability, we assessed the percentage of voxels with an orientation difference less than 15° between the estimation set and the left-out test run. Across participants, we observed significant temporal stability (percentage of stable voxels > 50% in 27 of 46 participants, one-sample *t*-test: $t(45) = 2.78$, $p = .01$). **f** Across participants, this temporal stability correlated significantly positively with the magnitude of the hexadirectional modulation (Pearson $r(44) = .86$, $p < .001$). **g** There was no significant correlation between the magnitude of the hexadirectional modulation and task performance (Pearson $r(44) = -.08$, $p = .59$). **h** Sampling of directions after median split of

trajectories according to their mean value (repeated measures ANOVA for interaction between value condition and direction: $F(35,1575) = 3.56$, $p < .001$). **i** Hexadirectional modulation in the significant entorhinal cluster, separately for the high- and low-value condition. While the analysis suggested no difference between the two conditions (related-samples *t*-test: $t(45) = -1.30$, $p = .19$), it suggested a hexadirectional modulation effect only in the low-value condition (one-sided one-sample *t*-tests; low-value: $t(45) = 2.06$, $p = .02$; high-value: $t(45) = 0.30$, $p = .38$). **j** There was no significant grid-like hexadirectional modulation of activity aligned to the respective grid orientation of two vmPFC ROIs, the first one defined based on the value difference effect in vmPFC in our study (left; one-sided one-sample *t*-tests; $t(45) = -0.34$, $p = .63$) and the second one defined based on the hexadirectional effect in vmPFC reported by Constantinescu et al. (2016)[2] (right; $t(45) = -0.94$, $p = .82$). **b,e,i,j** Dots represent data from $n = 46$ participants; boxplots show median and upper/lower quartile with whiskers extending to the most extreme data point within 1.5 interquartile ranges above/below the quartiles; black circles with error bars correspond to mean ± SEM; distributions depict probability density functions of data points. **d,f,g** Dots represent data from $n = 46$ participants; line represents linear regression line, with shaded regions as the 95% confidence interval. If not indicated otherwise, statistical tests were two-sided. * $p < .05$, ** $p < .01$. Source data are provided as a Source Data file.

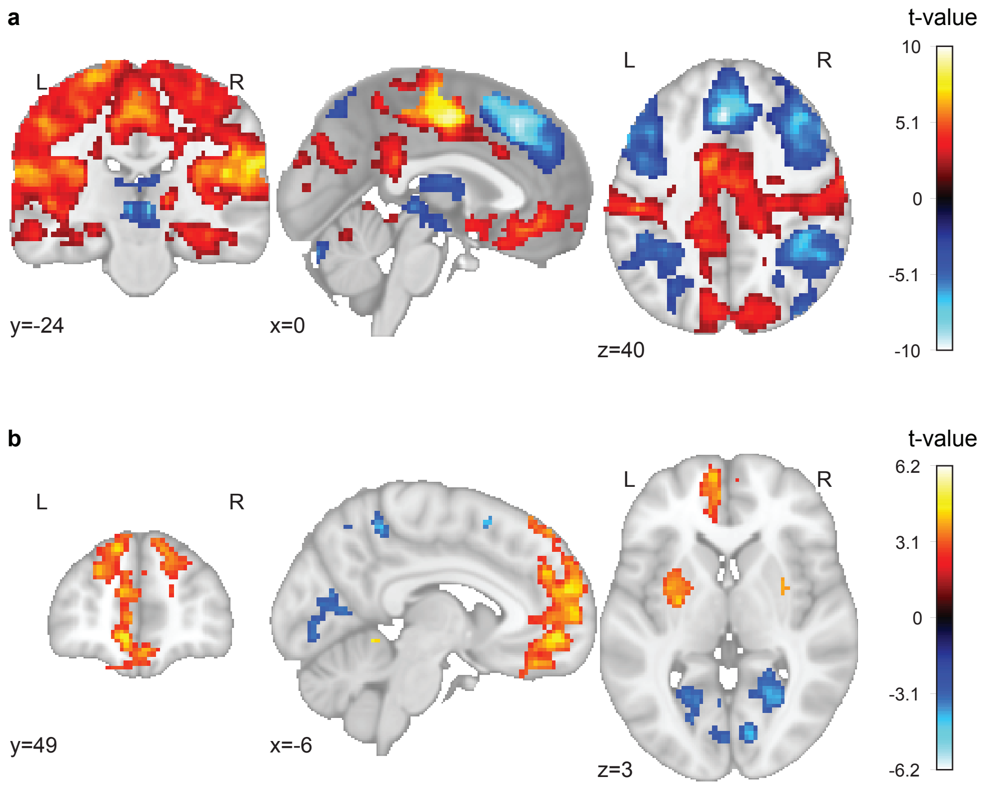

Supplementary Fig. 5 | Prospective value difference effects after controlling for reaction time. a Modulation of activity by the difference between model-derived chosen vs. unchosen value during choices after controlling for reaction time. Clusters depicted survive whole-brain correction (two-sided non-parametric permutation test with TFCE and p_{FWE} < .05). Statistical image is displayed on the MNI template. **b** Modulation of activity by the prospective component of the value difference during choices after controlling for reaction time. The prospective component refers to the influence of values estimated by the prospective Rescorla-Wagner model over values estimated by the original (non-prospective) Rescorla-Wagner model. Clusters depicted survive whole-brain correction (two-sided non-parametric permutation test with TFCE and p_{FWE} < .05). Statistical image is displayed on the MNI template. Source data are provided as a Source Data file.

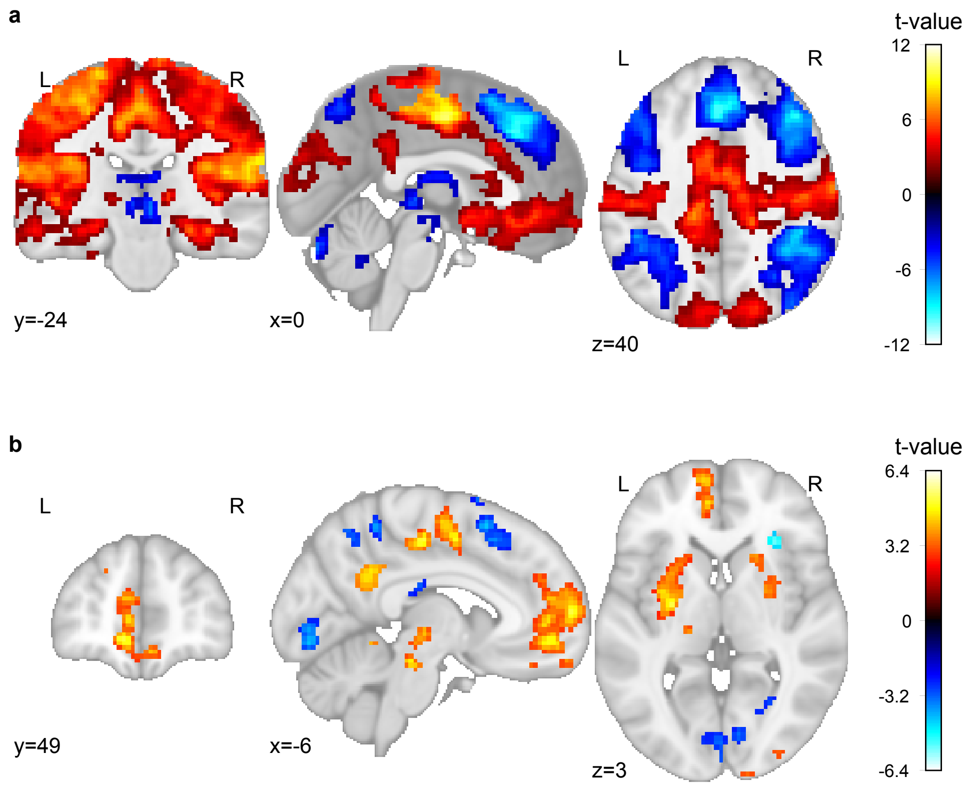

Supplementary Fig. 6 | Prospective value difference effects when including only correct trials. a Modulation of activity by the difference between model-derived chosen vs. unchosen value during choices when including only correct trials. Clusters depicted survive whole-brain correction (two-sided non-parametric permutation test with TFCE and $p_{FWE} < .05$). Statistical image is displayed on the MNI template. **b** Modulation of activity by the prospective component of the value difference during choices when including only correct trials. The prospective component refers to the influence of values estimated by the prospective Rescorla-Wagner model over values estimated by the original (non-prospective) Rescorla-Wagner model. Clusters depicted survive whole-brain correction (two-sided non-parametric permutation test with TFCE and $p_{FWE} < .05$). Statistical image is displayed on the MNI template. Source data are provided as a Source Data file.

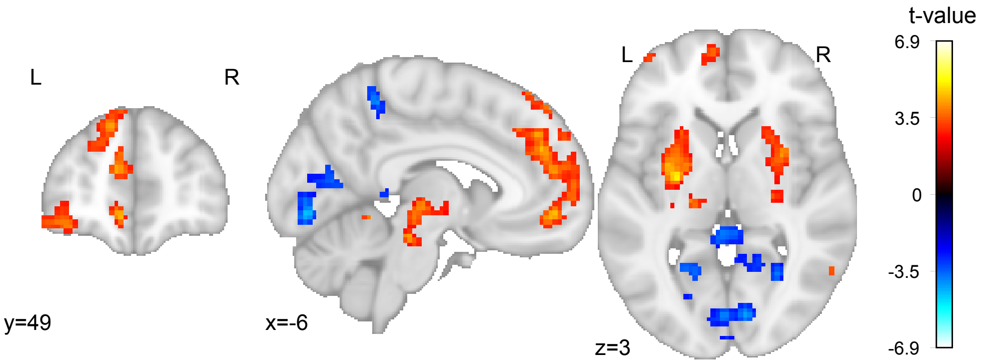

Supplementary Fig. 7 | Prospective value difference effects after controlling for the distance between the choice location and the 45°-diagonal. Modulation of activity by the prospective component of the value difference during choices after controlling for the distance between the choice location and the 45°-diagonal. The prospective component refers to the influence of values estimated by the prospective Rescorla-Wagner model over values estimated by the original (non-prospective) Rescorla-Wagner model. Clusters depicted survive whole-brain correction (two-sided non-parametric permutation test with TFCE and $p_{FWE} < .05$). Statistical image is displayed on the MNI template. Source data are provided as a Source Data file.

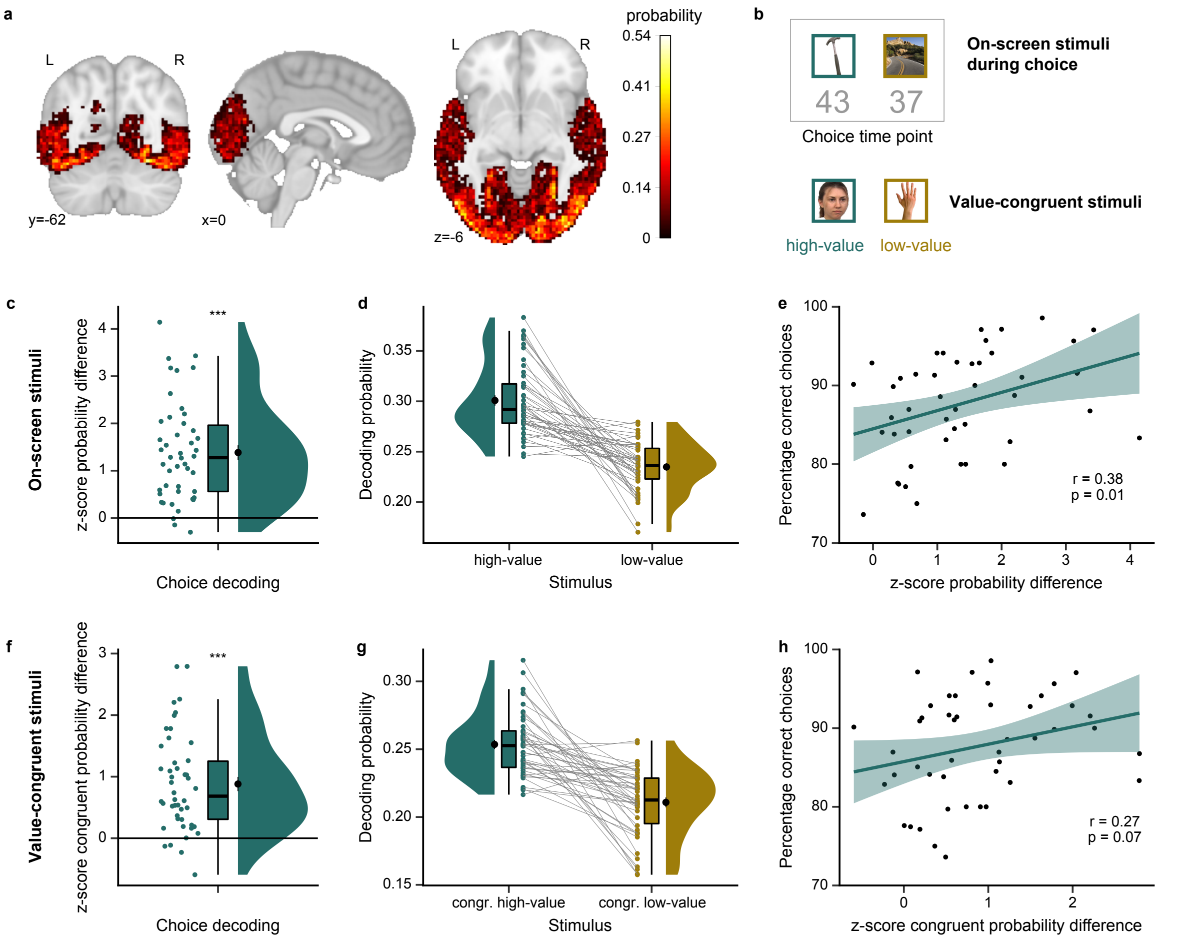

Supplementary Fig. 8 | Occipital-temporal representations of choice options (see also control analyses in Supplementary Fig. 9 and 10). To make prospective decisions in the absence of direct experience, stimulus values need to be constructed based on past experience. We assumed that this construction process might involve the sensory representations of the respective choice options. We thus hypothesized that we might be able to index this construction process by assessing the stimulus representations at the choice point, with the idea that the identity of the option associated with the objectively higher value might be represented more strongly than that of the option associated with the objectively lower value, in particular in participants who performed the task well (see Methods for analysis details). **a** To test this hypothesis, we leveraged neural responses to category-specific stimuli (faces, tools, scenes, body parts), which are known to activate category-selective regions of the occipital-temporal cortex. Using data from an independent picture viewing task (PVT) which took place before the prospective decision making task, we trained a decoder (support vector classifier) on occipital-temporal cortex voxels to distinguish neural activation patterns of the four category-specific stimuli (performance in PVT cover task: M = 94.60 %, SD = 9.87 %). The map depicts the probability of occipital-temporal cortex voxels in MNI space to be included in the mask across participants. Note that the decoding analysis was performed using participant-specific masks in native space, which were created based on anatomical and functional information (M = 2235 voxels). Control analyses showed that decoding accuracy within the PVT was well above chance level (M = 80.24 %, SD = 7.09 %, chance level = 25 %). **b** Example for stimuli at a choice time point in the prospective decision making task. Top: Stimuli which are on-screen during the choice. Objective values of the stimuli at the choice time point are shown in grey for visualization (not shown to participants). Below: Value-congruent stimuli which are not presented on-screen during the choice. We applied the trained decoder to neural activation patterns of choice time points. We compared decoding probabilities for the high- vs. low-value stimulus, separately for on-screen stimuli (c-e) and value-congruent off-screen stimuli (f-h). **c** Z-scores for the decoding probability difference for the on-screen high- vs. low-value stimuli based on decoding permutation test (see Methods). Probabilities for the high-value stimulus were significantly higher than for the low-value stimulus (one-sample t-test: $t(45) = 8.92$, $p < .001$). **d** Visualization of the effect in c, showing the probabilities the decoder assigned to the stimuli (before the permutation test). **e** The high- vs. low-value difference score correlated significantly positively with performance (Pearson $r(44) = .38$, $p = .01$). **f** Z-scores for the decoding probability difference for the value-congruent off-screen high- vs. low-value stimuli based on decoding permutation test (see Methods). Probabilities for the congruent high-value stimulus were significantly higher than for the congruent low-value stimulus (one-sample t-test: $t(45) = 7.54$, $p < .001$). **g** Visualization of the effect in f, showing the probabilities the decoder assigned to the stimuli (before the permutation test). **h** The correlation between the congruent high- vs. low-value difference score and performance was not significant

(Pearson $r(44) = .27$, $p = .07$). **c,d,f,g** Dots represent data from n = 46 participants; boxplots show median and upper/lower quartile with whiskers extending to the most extreme data point within 1.5 interquartile ranges above/below the quartiles; black circles with error bars correspond to mean ± SEM; distributions depict probability density functions of data points. **e,h** Dots represent data from n = 46 participants; line represents linear regression line, with shaded regions as the 95% confidence interval. All statistical tests were two-sided. *** $p < .001$. Source data are provided as a Source Data file. Stimuli taken from publicly available stimulus datasets (see Article Methods)[3–8].

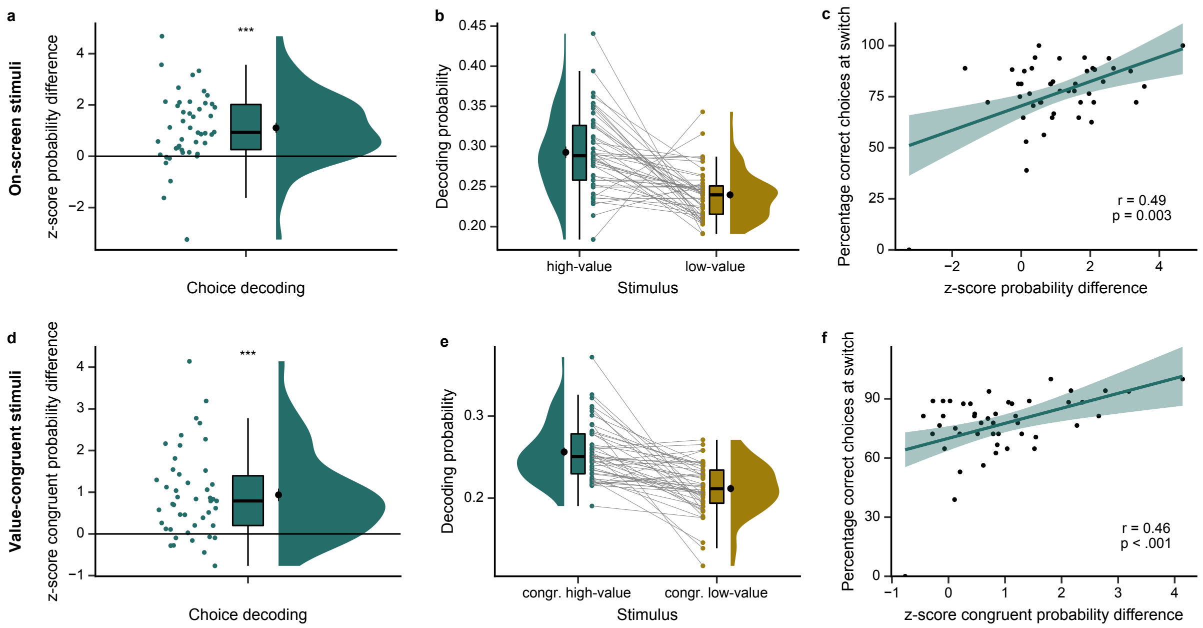

Supplementary Fig. 9 | Occipital-temporal representations of choice options when using only switch time points. Control analysis using only those choices which sampled the switch time point. While we compared on-screen and off-screen congruent stimuli separately (Supplementary Fig. 8), the temporal proximity of their presentations during time points within a trajectory might render disentangling their effects difficult. To control for the temporal proximity to some extent, we repeated the analysis using only those choices which sampled the switch time point. In this case, the direction of the effect during choice (high-value vs. low-value, especially for the comparison of the congruent stimuli) should be different from the direction of the effect at the time point before the switch (pre). Logic of the figures is the same as in Supplementary Fig. 8. **a** Z-scores for the probability difference for the on-screen high- vs. low-value stimuli based on decoding permutation test (see Methods). Probabilities for the high-value stimulus were significantly higher than for the low-value stimulus (one-sample *t*-test: $t(45) = 5.42$, $p < .001$). **b** Visualization of the effect in a, showing the probabilities the decoder assigned to the stimuli (before the permutation test). **c** Correlation between the high- vs. low-value difference score and performance at the switch time point (Pearson $r(44) = .49$, $p = .003$; after exclusion of two outliers: $r(42) = .17$, $p = .28$). **d** Z-scores for the probability difference for the congruent high- vs. low-value stimuli based on decoding permutation test (see Methods). Probabilities for the congruent high-value stimulus were significantly higher than for the congruent low-value stimulus (one-sample *t*-test: $t(45) = 6.17$, $p < .001$). **e** Visualization of the effect in d, showing the probabilities the decoder assigned to the stimuli (before the permutation test). **f** The congruent high-value vs. low-value difference score correlated significantly positively with performance at the switch time point (Pearson $r(44) = .46$, $p < .001$; after exclusion of two outliers: $r(42) = .34$, $p = .02$). **a,b,d,e** Dots represent data from n = 46 participants; boxplots show median and upper/lower quartile with whiskers extending to the most extreme data point within 1.5 interquartile ranges above/below the quartiles; black circles with error bars correspond to mean ± SEM; distributions depict probability density functions of data points. **c,f** Dots represent data from n = 46 participants; line represents linear regression line, with shaded regions as the 95% confidence interval. All statistical tests were two-sided. *** $p < .001$. Source data are provided as a Source Data file.

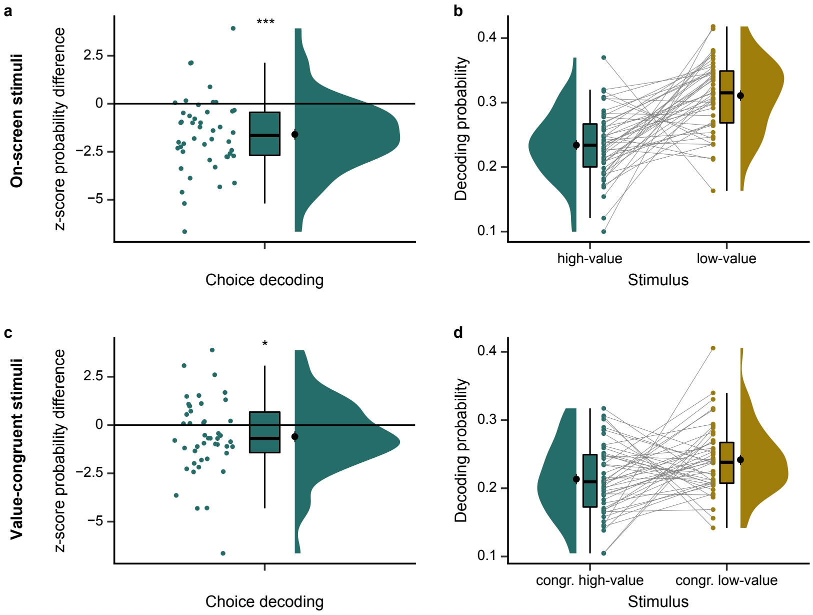

Supplementary Fig. 10 | Occipital-temporal representations of choice options when using only incorrect trials. Control analysis using only incorrect trials. Given that participants performed the task very well (*M* = 87.70 %), the stronger representation of the high-value option compared to the low-value option might be driven by selective attention towards the chosen option. To disentangle a value from an attention / choice effect, we conducted the analysis using only incorrectly answered trials (note the very low number of available incorrect trials). In this case, a stronger representation of the high vs. low value option would suggest a value effect while the opposite pattern, a stronger representation of the low-value option, would suggest an attention / choice effect. Indeed, this control analysis suggested that the stronger representation of the high-value vs. the low-value option is rather driven by selective attention towards the chosen option. Logic of the figures is the same as in Supplementary Fig. 8. **a** Z-scores for the decoding probability difference for the on-screen high- vs. low-value stimuli based on decoding permutation test (see Methods). Probabilities for the high-value stimulus were significantly lower than for the low-value stimulus (one-sample t-test: $t(45) = -5.57$, $p < .001$). **b** Visualization of the effect in a, showing the probabilities the decoder assigned to the stimuli (before the permutation test). **c** Z-scores for the decoding probability difference for the value-congruent off-screen high- vs. low-value stimuli based on decoding permutation test (see Methods). Probabilities for the congruent high-value stimulus were significantly lower than for the congruent low-value stimulus (one-sample t-test: $t(45) = -2.08$, $p = .04$). **d** Visualization of the effect in c, showing the probabilities the decoder assigned to the stimuli (before the permutation test). **a-d** Dots represent data from n = 46 participants; boxplots show median and upper/lower quartile with whiskers extending to the most extreme data point within 1.5 interquartile ranges above/below the quartiles; black circles with error bars correspond to mean ± SEM; distributions depict probability density functions of data points. All statistical tests were two-sided. * $p < .05$, *** $p < .001$. Source data are provided as a Source Data file.

Supplementary Tables

Supplementary Table 1: Exploratory whole-brain clusters of the hexadirectional modulation effect

No clusters survived family-wise error correction in the whole-brain analysis (one-sided non-parametric permutation test with TFCE and $p_{FWE} < .05$). This table lists clusters at an uncorrected threshold of $p < .001$ with a minimum of 10 voxels. Table lists MNI coordinates (X, Y, Z), statistical T values and atlas labels of peak voxels of the clusters. Atlas labels are based on the Harvard-Oxford Cortical Structural Atlas (HOCSA), Harvard-Oxford Subcortical Structural Atlas (HOSSA) and Juelich Histological Atlas (JHA) provided by FSL. If no label was found for a given atlas, the atlas is not listed. Subclusters are denoted by letters after the cluster ID.

**Cluster ID**	**X**	**Y**	**Z**	**T value**	**Cluster Size (voxels)**	**Atlas label**
1	-9.0	-11.0	9.0	4.25	12	HOSSA: 100% Left Thalamus
						JHA: 1% WM Corticospinal tract L
2	18.0	-6.0	-26.0	4.17	30	HOCSA: 66% Parahippocampal Gyrus, anterior division
						HOSSA: 66% Right Cerebral Cortex, 24% Right Hippocampus, 8% Right Amygdala, 2% Right Cerebral White Matter
						JHA: 68% GM Hippocampus entorhinal cortex R, 27% GM Hippocampus subiculum R, 16% GM Hippocampus hippocampal-amygdaloid transition area R, 5% GM Hippocampus cornu ammonis

						R, 2% GM Amygdala_superficial group R, 1% GM Amygdala_laterobasal group R
3	6.0	-26.0	-38.0	3.95	12	HOSSA:<br>100% Brain-Stem
4	-27.0	-3.0	-28.0	3.91	26	HOCSA:<br>9% Parahippocampal Gyrus, anterior division, 1% Temporal Pole
						HOSSA:<br>50% Left Amygdala, 27% Left Cerebral White Matter, 12% Left Cerebral Cortex, 11% Left Hippocampus
						JHA:<br>92% GM Amygdala_laterobasal group L, 5% GM Amygdala_superficial group L, 4% GM Hippocampus subiculum L, 2% WM Optic radiation L, 2% GM Hippocampus cornu ammonis L
4a	-22.0	-1.0	-38.0	3.47		HOCSA:<br>69% Parahippocampal Gyrus, anterior division, 6% Temporal Fusiform Cortex, anterior division, 3% Temporal Fusiform Cortex, posterior division, 1% Temporal Pole
						HOSSA:<br>85% Left Cerebral Cortex, 2% Left Cerebral White Matter
						JHA:

						98% GM Hippocampus entorhinal cortex L
5	13.0	-21.0	-31.0	3.59	14	HOSSA:<br>99% Brain-Stem

Supplementary Table 2: Significant clusters of the value difference effect

Clusters surviving family-wise error correction in the whole-brain analysis (two-sided non-parametric permutation test with TFCE and $p_{FWE} < .05$). Table lists MNI coordinates (X, Y, Z), statistical T values and atlas labels of peak voxels of the clusters. Atlas labels are based on the Harvard-Oxford Cortical Structural Atlas (HOCSA), Harvard-Oxford Subcortical Structural Atlas (HOSSA) and Juelich Histological Atlas (JHA) provided by FSL. If no label was found for a given atlas, the atlas is not listed. Subclusters are denoted by letters after the cluster ID.

Cluster ID	X	Y	Z	T value	Cluster Size (voxels)	Atlas label
**Positive effect**						
1	-34.0	2.0	14.0	10.95	32064	HOCSA:<br>37% Central Opercular Cortex, 15% Insular Cortex, 1% Frontal Operculum Cortex
						HOSSA:<br>73% Left Cerebral Cortex, 27% Left Cerebral White Matter
1a	-4.0	-3.0	46.0	10.39		HOCSA:<br>46% Cingulate Gyrus, anterior division, 40% Juxtapositional Lobule Cortex (formerly Supplementary Motor Cortex)
						HOSSA:<br>87% Left Cerebral Cortex, 12% Left Cerebral White Matter
						JHA:<br>42% GM Premotor cortex BA6 L
1b	-49.0	-6.0	12.0	9.78		HOCSA:<br>49% Central Opercular Cortex, 2% Postcentral Gyrus, 1% Precentral Gyrus
						HOSSA:<br>51% Left Cerebral Cortex, 49% Left Cerebral White Matter
						JHA:<br>35% GM Secondary somatosensory cortex / Parietal operculum OP4 L, 9% GM Secondary somatosensory cortex / Parietal operculum OP1 L, 7% GM Broca's area BA44 L, 4% GM

						Primary auditory cortex TE1.0 L, 3% GM Secondary somatosensory cortex / Parietal operculum OP3 L
1c	50.0	-1.0	9.0	9.35		HOCSA:<br>59% Central Opercular Cortex, 2% Planum Polare, 1% Precentral Gyrus, 1% Inferior Frontal Gyrus, pars opercularis
						HOSSA:<br>63% Right Cerebral Cortex, 36% Right Cerebral White Matter
						JHA:<br>28% GM Secondary somatosensory cortex / Parietal operculum OP4 R, 20% GM Secondary somatosensory cortex / Parietal operculum OP3 R, 4% GM Inferior parietal lobule PFop R, 2% GM Primary auditory cortex TE1.2 R
2	23.0	-53.0	-56.0	7.21	83	No label
3	-22.0	-55.0	-58.0	6.21	31	No label
4	3.0	42.0	-8.0	5.99	1394	HOCSA:<br>63% Paracingulate Gyrus, 17% Frontal Medial Cortex, 16% Cingulate Gyrus, anterior division
						HOSSA:<br>91% Right Cerebral Cortex, 7% Left Cerebral Cortex, 1% Right Cerebral White Matter
4a	-4.0	32.0	-11.0	5.67		HOCSA:<br>33% Paracingulate Gyrus, 32% Subcallosal Cortex, 13% Cingulate Gyrus, anterior division, 9% Frontal Medial Cortex
						HOSSA:<br>90% Left Cerebral Cortex, 10% Left Cerebral White Matter, 0% Right Cerebral Cortex
4b	-7.0	27.0	-8.0	5.51		HOCSA:<br>63% Subcallosal Cortex, 3% Paracingulate Gyrus, 1% Cingulate Gyrus, anterior division
						HOSSA:

						69% Left Cerebral Cortex, 31% Left Cerebral White Matter<br>JHA:<br>4% WM Cingulum L, 1% WM Callosal body
4c	-29.0	32.0	-14.0	5.40		HOCSA:<br>46% Frontal Orbital Cortex, 19% Frontal Pole<br>HOSSA:<br>65% Left Cerebral Cortex, 35% Left Cerebral White Matter
5	33.0	34.0	-14.0	4.19	35	HOCSA:<br>48% Frontal Pole, 38% Frontal Orbital Cortex<br>HOSSA:<br>87% Right Cerebral Cortex, 13% Right Cerebral White Matter
5a	18.0	22.0	-14.0	2.79		HOCSA:<br>12% Frontal Orbital Cortex<br>HOSSA:<br>85% Right Cerebral White Matter, 15% Right Cerebral Cortex
6	-37.0	-11.0	-48.0	4.19	3	HOCSA:<br>11% Inferior Temporal Gyrus, anterior division, 8% Temporal Fusiform Cortex, posterior division, 8% Inferior Temporal Gyrus, posterior division, 6% Temporal Fusiform Cortex, anterior division<br>HOSSA:<br>38% Left Cerebral Cortex, 0% Left Cerebral White Matter
7	1.0	14.0	14.0	4.14	9	HOSSA:<br>51% Right Lateral Ventricle, 36% Right Cerebral White Matter, 9% Left Cerebral White Matter, 4% Left Lateral Ventricle, 0% Left Cerebral Cortex<br>JHA:<br>40% WM Callosal body
8	15.0	32.0	6.0	3.23	8	HOSSA:

						97% Right Cerebral White Matter, 3% Right Lateral Ventricle
						JHA:<br>97% WM Callosal body, 5% WM Cingulum R
**Negative effect**						
1	6.0	24.0	52.0	-10.28	11448	HOCSA:<br>52% Superior Frontal Gyrus, 10% Paracingulate Gyrus
						HOSSA:<br>79% Right Cerebral Cortex, 21% Right Cerebral White Matter
						JHA:<br>20% GM Premotor cortex BA6 R
1a	30.0	7.0	59.0	-9.56		HOCSA:<br>34% Middle Frontal Gyrus, 22% Superior Frontal Gyrus, 1% Precentral Gyrus
						HOSSA:<br>80% Right Cerebral Cortex, 10% Right Cerebral White Matter
1b	-2.0	29.0	39.0	-9.52		HOCSA:<br>61% Paracingulate Gyrus, 15% Superior Frontal Gyrus
						HOSSA:<br>86% Left Cerebral Cortex, 1% Right Cerebral Cortex, 0% Left Cerebral White Matter
1c	3.0	37.0	46.0	-9.49		HOCSA:<br>55% Superior Frontal Gyrus, 1% Paracingulate Gyrus
						HOSSA:<br>69% Right Cerebral Cortex, 9% Left Cerebral Cortex
						JHA:<br>1% GM Premotor cortex BA6 R
2	-37.0	-65.0	-28.0	-9.96	2796	No label
2a	-32.0	-65.0	-28.0	-9.93		No label
2b	30.0	-63.0	-28.0	-8.86		No label
2c	8.0	-78.0	-24.0	-8.52		HOCSA:

						3% Occipital Fusiform Gyrus, 2% Lingual Gyrus
						HOSSA:<br>5% Right Cerebral Cortex
3	43.0	-38.0	42.0	-9.09	3463	HOCSA:<br>41% Supramarginal Gyrus, posterior division, 10% Superior Parietal Lobule, 5% Angular Gyrus, 5% Postcentral Gyrus, 4% Supramarginal Gyrus, anterior division
						HOSSA:<br>68% Right Cerebral Cortex, 32% Right Cerebral White Matter
						JHA:<br>28% GM Anterior intra-parietal sulcus hIP3 R, 17% GM Anterior intra-parietal sulcus hIP2 R, 11% GM Superior parietal lobule 7PC R, 3% GM Anterior intra-parietal sulcus hIP1 R
3a	-47.0	-50.0	52.0	-8.25		HOCSA:<br>24% Supramarginal Gyrus, posterior division, 22% Angular Gyrus, 13% Superior Parietal Lobule, 2% Supramarginal Gyrus, anterior division, 1% Lateral Occipital Cortex, superior division
						HOSSA:<br>65% Left Cerebral Cortex, 25% Left Cerebral White Matter
						JHA:<br>40% GM Inferior parietal lobule PFm L, 32% GM Inferior parietal lobule PF L, 15% GM Anterior intra-parietal sulcus hIP2 L, 12% GM Inferior parietal lobule Pga L, 11% GM Anterior intra-parietal sulcus hIP1 L, 10% GM Superior parietal lobule 7PC L, 9% GM Anterior intra-parietal sulcus hIP3 L, 3% GM Superior parietal lobule 5L L
3b	48.0	-45.0	52.0	-8.04		HOCSA:<br>38% Supramarginal Gyrus, posterior division, 33% Angular Gyrus, 9% Superior Parietal Lobule

						HOSSA: 82% Right Cerebral Cortex, 17% Right Cerebral White Matter
						JHA: 77% GM Inferior parietal lobule PFm R, 13% GM Anterior intra-parietal sulcus hIP1 R, 12% GM Inferior parietal lobule Pga R, 12% GM Anterior intra-parietal sulcus hIP2 R, 2% GM Anterior intra-parietal sulcus hIP3 R, 1% GM Superior parietal lobule 7PC R
3c	38.0	-48.0	42.0	-7.39		HOCSA: 23% Superior Parietal Lobule, 18% Supramarginal Gyrus, posterior division, 15% Angular Gyrus
						HOSSA: 60% Right Cerebral Cortex, 39% Right Cerebral White Matter
						JHA: 45% GM Anterior intra-parietal sulcus hIP1 R, 29% GM Anterior intra-parietal sulcus hIP3 R, 20% GM Anterior intra-parietal sulcus hIP2 R
4	-12.0	2.0	-1.0	-8.09	1717	HOSSA: 60% Left Pallidum, 40% Left Cerebral White Matter
4a	8.0	7.0	9.0	-7.82		HOSSA: 57% Right Caudate, 41% Right Lateral Ventricle, 2% Right Cerebral White Matter
4b	10.0	2.0	14.0	-7.39		HOSSA: 68% Right Caudate, 30% Right Lateral Ventricle, 1% Right Cerebral White Matter, 1% Right Thalamus
4c	10.0	4.0	-1.0	-7.29		HOSSA: 90% Right Cerebral White Matter, 5% Right Caudate, 4% Right Pallidum, 0% Right Cerebral Cortex, 0% Right Thalamus, 0% Right Lateral Ventricle, 0% Right Accumbens
5	-19.0	42.0	-21.0	-6.0	19	HOCSA: 72% Frontal Pole, 3% Frontal Orbital Cortex

						HOSSA:<br>75% Left Cerebral Cortex, 20% Left Cerebral White Matter
6	-9.0	-58.0	-51.0	-5.73	5	No label

Supplementary Table 3: Significant clusters of the prospective value difference effect

Clusters surviving family-wise error correction in the whole-brain analysis (two-sided non-parametric permutation test with TFCE and $p_{FWE} < .05$). Table lists MNI coordinates (X, Y, Z), statistical T values and atlas labels of peak voxels of the clusters. Atlas labels are based on the Harvard-Oxford Cortical Structural Atlas (HOCSA), Harvard-Oxford Subcortical Structural Atlas (HOSSA) and Juelich Histological Atlas (JHA) provided by FSL. If no label was found for a given atlas, the atlas is not listed. Subclusters are denoted by letters after the cluster ID. In total, 96 clusters including subclusters were found. Table lists 10 clusters with the most extreme T values, separately for positive and negative effects.

Cluster ID	X	Y	Z	T value	Cluster Size (voxels)	Atlas label
**Positive effect**						
1	-34.0	-26.0	56.0	6.77	1029	HOCSA: 34% Postcentral Gyrus, 25% Precentral Gyrus
						HOSSA: 67% Left Cerebral Cortex, 32% Left Cerebral White Matter
						JHA: 44% WM Corticospinal tract L, 42% GM Primary motor cortex BA4p L, 28% GM Primary somatosensory cortex BA3b L, 28% GM Primary motor cortex BA4a L, 10% GM Primary somatosensory cortex BA1 L, 6% GM Primary somatosensory cortex BA2 L
1a	-32.0	-23.0	62.0	6.50		HOCSA: 34% Precentral Gyrus, 14% Postcentral Gyrus
						HOSSA: 55% Left Cerebral Cortex, 44% Left Cerebral White Matter
						JHA: 51% GM Premotor cortex BA6 L, 47% WM Corticospinal tract L, 32% GM Primary motor cortex BA4a L, 5% GM Primary somatosensory cortex BA3b L, 5% GM Primary

						somatosensory cortex BA1 L, 4% GM Primary motor cortex BA4p L
1b	-34.0	-33.0	66.0	6.39		HOCSA:<br>51% Postcentral Gyrus, 6% Superior Parietal Lobule, 3% Precentral Gyrus, 2% Supramarginal Gyrus, anterior division
						HOSSA:<br>66% Left Cerebral Cortex, 22% Left Cerebral White Matter
						JHA:<br>71% GM Primary somatosensory cortex BA1 L, 30% GM Primary motor cortex BA4a L, 29% GM Primary somatosensory cortex BA2 L, 27% GM Primary somatosensory cortex BA3b L, 12% GM Primary motor cortex BA4p L, 8% GM Superior parietal lobule 5L L, 5% GM Superior parietal lobule 7PC L, 1% WM Corticospinal tract L
1c	-54.0	-26.0	49.0	6.09		HOCSA:<br>61% Postcentral Gyrus, 17% Supramarginal Gyrus, anterior division
						HOSSA:<br>84% Left Cerebral Cortex, 11% Left Cerebral White Matter
						JHA:<br>58% GM Primary somatosensory cortex BA2 L, 56% GM Primary somatosensory cortex BA1 L, 31% GM Inferior parietal lobule PF L, 20% GM Inferior parietal lobule PFt L, 9% GM Anterior intra-parietal sulcus hIP2 L, 3% GM Primary somatosensory cortex BA3b L, 2% GM Inferior parietal lobule PFop L
2	45.0	-28.0	26.0	6.67	124	HOCSA:<br>31% Parietal Operculum Cortex, 10% Supramarginal Gyrus, anterior division, 1% Supramarginal Gyrus, posterior division
						HOSSA:

						56% Right Cerebral White Matter, 44% Right Cerebral Cortex
						JHA:<br>40% GM Secondary somatosensory cortex / Parietal operculum OP1 R, 34% GM Inferior parietal lobule PFop R, 24% GM Inferior parietal lobule PFcm R, 4% GM Anterior intra-parietal sulcus hIP2 R
3	-49.0	-23.0	22.0	6.38	107	HOCSA:<br>31% Central Opercular Cortex, 15% Parietal Operculum Cortex, 4% Postcentral Gyrus, 1% Supramarginal Gyrus, anterior division
						HOSSA:<br>53% Left Cerebral Cortex, 47% Left Cerebral White Matter
						JHA:<br>72% GM Secondary somatosensory cortex / Parietal operculum OP1 L, 19% GM Inferior parietal lobule PFop L, 9% GM Secondary somatosensory cortex / Parietal operculum OP4 L, 7% GM Inferior parietal lobule PFcm L, 3% GM Primary auditory cortex TE1.0 L
3a	-44.0	-33.0	22.0	4.07		HOCSA:<br>60% Parietal Operculum Cortex, 3% Supramarginal Gyrus, posterior division, 1% Central Opercular Cortex, 1% Superior Temporal Gyrus, posterior division
						HOSSA:<br>67% Left Cerebral Cortex, 31% Left Cerebral White Matter
						JHA:<br>63% GM Inferior parietal lobule PFcm L, 30% GM Secondary somatosensory cortex / Parietal operculum OP1 L, 3% GM Inferior parietal lobule PFop L, 1% GM Inferior parietal lobule PF L
4	50.0	-63.0	-6.0	6.06	77	HOCSA:

						44% Lateral Occipital Cortex, inferior division, 11% Inferior Temporal Gyrus, temporooccipital part, 8% Middle Temporal Gyrus, temporooccipital part, 2% Occipital Fusiform Gyrus
						HOSSA:<br>67% Right Cerebral Cortex, 33% Right Cerebral White Matter
						JHA:<br>8% GM Visual cortex V5 R
5	-39.0	-1.0	16.0	5.78	1093	HOCSA:<br>57% Central Opercular Cortex, 10% Insular Cortex
						HOSSA:<br>78% Left Cerebral Cortex, 22% Left Cerebral White Matter
5a	-29.0	7.0	-6.0	5.56		HOSSA:<br>73% Left Cerebral White Matter, 27% Left Putamen, 0% Left Cerebral Cortex
						JHA:<br>58% WM Inferior occipito-frontal fascicle L, 12% WM Uncinate fascicle L
5b	-32.0	-6.0	2.0	5.52		HOSSA:<br>61% Left Putamen, 39% Left Cerebral White Matter, 0% Left Cerebral Cortex
5c	-22.0	-13.0	-18.0	5.09		HOSSA:<br>91% Left Hippocampus, 2% Left Amygdala
						JHA:<br>80% GM Hippocampus cornu ammonis L, 42% GM Hippocampus subiculum L, 34% GM Hippocampus dentate gyrus L, 14% GM Amygdala_superficial group L, 13% GM Hippocampus hippocampal-amygdaloid transition area L, 13% GM Hippocampus entorhinal cortex L, 11% GM Amygdala_laterobasal group L, 4% GM Amygdala_centromedial group L

6	-54.0	-65.0	39.0	5.77	273	HOCSA:<br>38% Lateral Occipital Cortex, superior division, 1% Angular Gyrus
						HOSSA:<br>48% Left Cerebral Cortex, 0% Left Cerebral White Matter
6a	-54.0	-65.0	34.0	5.56		HOCSA:<br>74% Lateral Occipital Cortex, superior division, 5% Angular Gyrus
						HOSSA:<br>85% Left Cerebral Cortex, 1% Left Cerebral White Matter
						JHA:<br>32% GM Inferior parietal lobule PGp L, 16% GM Inferior parietal lobule Pga L, 12% GM Inferior parietal lobule PFm L
6b	-52.0	-55.0	29.0	4.31		HOCSA:<br>49% Angular Gyrus, 14% Supramarginal Gyrus, posterior division, 5% Lateral Occipital Cortex, superior division
						HOSSA:<br>70% Left Cerebral Cortex, 29% Left Cerebral White Matter
						JHA:<br>47% GM Inferior parietal lobule Pga L, 31% GM Inferior parietal lobule PFm L, 20% GM Inferior parietal lobule PF L
7	-7.0	52.0	-8.0	5.75	1423	HOCSA:<br>53% Frontal Medial Cortex, 24% Paracingulate Gyrus, 13% Frontal Pole
						HOSSA:<br>91% Left Cerebral Cortex, 9% Left Cerebral White Matter
7a	-19.0	59.0	26.0	5.20		HOCSA:<br>75% Frontal Pole
						HOSSA:

						79% Left Cerebral Cortex, 15% Left Cerebral White Matter
7b	-14.0	54.0	34.0	4.98		HOCSA:<br>79% Frontal Pole
						HOSSA:<br>82% Left Cerebral Cortex, 9% Left Cerebral White Matter
7c	-4.0	59.0	19.0	4.82		HOCSA:<br>53% Frontal Pole, 19% Superior Frontal Gyrus, 8% Paracingulate Gyrus
						HOSSA:<br>91% Left Cerebral Cortex, 4% Left Cerebral White Matter
8	28.0	-8.0	-18.0	5.16	539	HOSSA:<br>48% Right Amygdala, 34% Right Hippocampus, 2% Right Cerebral White Matter
						JHA:<br>79% GM Amygdala_laterobasal group R, 74% GM Hippocampus cornu ammonis R, 15% GM Hippocampus dentate gyrus R, 5% GM Amygdala_superficial group R, 3% GM Hippocampus subiculum R, 1% GM Amygdala_centromedial group R
8a	18.0	-11.0	-14.0	4.79		HOSSA:<br>78% Right Amygdala, 12% Right Hippocampus, 5% Right Cerebral White Matter, 0% Right Cerebral Cortex
						JHA:<br>35% GM Amygdala_superficial group R, 23% GM Hippocampus subiculum R, 22% GM Hippocampus hippocampal-amygdaloid transition area R, 14% WM Corticospinal tract R, 14% GM Amygdala_laterobasal group R, 12% GM Hippocampus cornu ammonis R, 8% GM Amygdala_centromedial group R, 6% GM Hippocampus entorhinal cortex R, 2% WM Acoustic radiation R

8b	25.0	12.0	-1.0	4.73		HOSSA:<br>96% Right Putamen, 4% Right Cerebral White Matter
8c	40.0	-30.0	-21.0	4.22		HOCSA:<br>53% Temporal Fusiform Cortex, posterior division, 11% Inferior Temporal Gyrus, posterior division, 3% Inferior Temporal Gyrus, temporooccipital part, 2% Temporal Occipital Fusiform Cortex
						HOSSA:<br>69% Right Cerebral Cortex, 30% Right Cerebral White Matter
						JHA:<br>2% GM Hippocampus cornu ammonis R, 1% WM Optic radiation R
9	-2.0	-48.0	32.0	4.90	57	HOCSA:<br>84% Cingulate Gyrus, posterior division, 10% Precuneous Cortex
						HOSSA:<br>96% Left Cerebral Cortex, 1% Left Cerebral White Matter, 1% Right Cerebral Cortex
9a	-9.0	-53.0	29.0	4.59		HOCSA:<br>32% Cingulate Gyrus, posterior division, 11% Precuneous Cortex
						HOSSA:<br>57% Left Cerebral White Matter, 43% Left Cerebral Cortex
						JHA:<br>3% WM Callosal body
10	-7.0	-23.0	49.0	4.85	327	HOCSA:<br>54% Precentral Gyrus, 15% Cingulate Gyrus, posterior division, 3% Cingulate Gyrus, anterior division, 3% Juxtapositional Lobule Cortex (formerly Supplementary Motor Cortex)
						HOSSA:<br>81% Left Cerebral Cortex, 19% Left Cerebral White Matter
						JHA:

						40% GM Premotor cortex BA6 L, 34% GM Primary motor cortex BA4a L, 4% WM Corticospinal tract L, 3% GM Superior parietal lobule 5M L
10a	-4.0	-6.0	56.0	4.77		HOCSA:<br>78% Juxtapositional Lobule Cortex (formerly Supplementary Motor Cortex)
						HOSSA:<br>81% Left Cerebral Cortex, 17% Left Cerebral White Matter
						JHA:<br>78% GM Premotor cortex BA6 L
10b	-7.0	-11.0	59.0	4.70		HOCSA:<br>43% Juxtapositional Lobule Cortex (formerly Supplementary Motor Cortex), 6% Precentral Gyrus
						HOSSA:<br>52% Left Cerebral Cortex, 48% Left Cerebral White Matter
						JHA:<br>96% GM Premotor cortex BA6 L, 3% WM Corticospinal tract L
10c	8.0	-3.0	56.0	4.15		HOCSA:<br>44% Juxtapositional Lobule Cortex (formerly Supplementary Motor Cortex), 2% Precentral Gyrus, 1% Cingulate Gyrus, anterior division
						HOSSA:<br>50% Right Cerebral White Matter, 50% Right Cerebral Cortex
						JHA:<br>61% GM Premotor cortex BA6 R
**Negative effect**						
1	30.0	29.0	4.0	-6.77	23	HOCSA:<br>11% Frontal Orbital Cortex, 7% Insular Cortex, 6% Inferior Frontal Gyrus, pars triangularis, 3% Frontal Operculum Cortex
						HOSSA:

						64% Right Cerebral White Matter, 36% Right Cerebral Cortex
2	6.0	-65.0	52.0	-6.51	1337	HOCSA:<br>66% Precuneous Cortex, 2% Lateral Occipital Cortex, superior division
						HOSSA:<br>70% Right Cerebral Cortex, 30% Right Cerebral White Matter
						JHA:<br>38% GM Superior parietal lobule 7P R, 32% GM Superior parietal lobule 7A R, 5% GM Superior parietal lobule 7M R
2a	-2.0	-63.0	54.0	-5.35		HOCSA:<br>83% Precuneous Cortex
						HOSSA:<br>85% Left Cerebral Cortex, 6% Left Cerebral White Matter, 1% Right Cerebral Cortex
						JHA:<br>42% GM Superior parietal lobule 7A L, 33% GM Superior parietal lobule 7P L
2b	-24.0	-53.0	42.0	-5.27		HOCSA:<br>24% Superior Parietal Lobule, 5% Lateral Occipital Cortex, superior division, 4% Supramarginal Gyrus, posterior division, 2% Angular Gyrus
						HOSSA:<br>64% Left Cerebral White Matter, 36% Left Cerebral Cortex
						JHA:<br>17% GM Anterior intra-parietal sulcus hIP3 L, 10% GM Anterior intra-parietal sulcus hIP1 L, 1% GM Superior parietal lobule 7A L
2c	10.0	-43.0	46.0	-5.25		HOCSA:<br>56% Precuneous Cortex, 16% Cingulate Gyrus, posterior division, 2% Postcentral Gyrus
						HOSSA:

						76% Right Cerebral Cortex, 24% Right Cerebral White Matter
						JHA:<br>20% GM Superior parietal lobule 5Ci R, 5% GM Superior parietal lobule 5M R
3	1.0	17.0	49.0	-6.41	351	HOCSA:<br>54% Paracingulate Gyrus, 7% Superior Frontal Gyrus, 2% Cingulate Gyrus, anterior division
						HOSSA:<br>69% Right Cerebral Cortex, 7% Left Cerebral Cortex, 0% Right Cerebral White Matter
						JHA:<br>29% GM Premotor cortex BA6 R
3a	3.0	29.0	46.0	-4.15		HOCSA:<br>31% Superior Frontal Gyrus, 27% Paracingulate Gyrus
						HOSSA:<br>67% Right Cerebral Cortex, 9% Left Cerebral Cortex
						JHA:<br>3% GM Premotor cortex BA6 R
3b	8.0	32.0	44.0	-3.79		HOCSA:<br>31% Superior Frontal Gyrus, 11% Paracingulate Gyrus
						HOSSA:<br>57% Right Cerebral Cortex, 43% Right Cerebral White Matter
						JHA:<br>8% GM Premotor cortex BA6 R
4	1.0	-88.0	-11.0	-6.33	732	HOCSA:<br>45% Lingual Gyrus, 6% Occipital Pole, 3% Intracalcarine Cortex, 1% Occipital Fusiform Gyrus
						HOSSA:<br>32% Right Cerebral Cortex, 26% Left Cerebral Cortex, 4% Right Cerebral White Matter, 0% Left Cerebral White Matter
						JHA:

						4% GM Visual cortex V1 BA17 R
4a	8.0	-80.0	-8.0	-5.38		HOCSA:<br>71% Lingual Gyrus, 6% Occipital Fusiform Gyrus, 1% Intracalcarine Cortex
						HOSSA:<br>78% Right Cerebral Cortex, 18% Right Cerebral White Matter, 0% Left Cerebral Cortex
						JHA:<br>56% GM Visual cortex V1 BA17 R, 52% GM Visual cortex V2 BA18 R, 12% WM Optic radiation R, 6% GM Visual cortex V3V R
4b	-12.0	-73.0	16.0	-4.24		HOCSA:<br>24% Intracalcarine Cortex, 9% Cuneal Cortex, 3% Supracalcarine Cortex, 2% Precuneous Cortex
						HOSSA:<br>59% Left Cerebral White Matter, 41% Left Cerebral Cortex
						JHA:<br>40% GM Visual cortex V1 BA17 L, 18% WM Optic radiation L, 10% GM Visual cortex V2 BA18 L
4c	13.0	-65.0	9.0	-4.18		HOCSA:<br>61% Intracalcarine Cortex, 4% Lingual Gyrus, 2% Supracalcarine Cortex
						HOSSA:<br>67% Right Cerebral Cortex, 33% Right Cerebral White Matter
						JHA:<br>74% GM Visual cortex V1 BA17 R, 33% GM Visual cortex V2 BA18 R, 32% WM Optic radiation R, 10% GM Visual cortex V3V R, 1% GM Visual cortex V4 R
5	30.0	-60.0	52.0	-5.93	517	HOCSA:<br>44% Lateral Occipital Cortex, superior division, 11% Superior Parietal Lobule, 7% Angular Gyrus
						HOSSA:

						66% Right Cerebral Cortex, 29% Right Cerebral White Matter
						JHA:<br>37% GM Anterior intra-parietal sulcus hIP3 R, 28% GM Superior parietal lobule 7A R, 10% GM Anterior intra-parietal sulcus hIP1 R, 2% GM Superior parietal lobule 7PC R, 1% GM Superior parietal lobule 7P R
5a	33.0	-53.0	44.0	-5.0		HOCSA:<br>25% Superior Parietal Lobule, 21% Angular Gyrus, 8% Lateral Occipital Cortex, superior division, 2% Supramarginal Gyrus, posterior division
						HOSSA:<br>59% Right Cerebral Cortex, 41% Right Cerebral White Matter
						JHA:<br>25% GM Anterior intra-parietal sulcus hIP3 R, 11% GM Anterior intra-parietal sulcus hIP1 R, 6% GM Anterior intra-parietal sulcus hIP2 R, 2% GM Superior parietal lobule 7A R
5b	45.0	-43.0	49.0	-4.95		HOCSA:<br>41% Supramarginal Gyrus, posterior division, 18% Angular Gyrus, 8% Superior Parietal Lobule, 2% Postcentral Gyrus, 1% Supramarginal Gyrus, anterior division
						HOSSA:<br>71% Right Cerebral Cortex, 27% Right Cerebral White Matter
						JHA:<br>42% GM Inferior parietal lobule PFm R, 15% GM Anterior intra-parietal sulcus hIP2 R, 15% GM Anterior intra-parietal sulcus hIP1 R, 14% GM Anterior intra-parietal sulcus hIP3 R, 5% GM Inferior parietal lobule Pga R, 4% GM Superior parietal lobule 7PC R, 2% GM Inferior parietal lobule PF R

5c	50.0	-35.0	52.0	-4.88		HOCSA:<br>30% Supramarginal Gyrus, anterior division, 28% Supramarginal Gyrus, posterior division, 13% Postcentral Gyrus, 2% Superior Parietal Lobule, 1% Angular Gyrus
						HOSSA:<br>81% Right Cerebral Cortex, 15% Right Cerebral White Matter
						JHA:<br>39% GM Inferior parietal lobule PFt R, 28% GM Inferior parietal lobule PFm R, 25% GM Primary somatosensory cortex BA2 R, 19% GM Anterior intra-parietal sulcus hIP2 R, 10% GM Superior parietal lobule 7PC R, 2% GM Primary somatosensory cortex BA1 R
6	30.0	12.0	56.0	-5.38	72	HOCSA:<br>35% Middle Frontal Gyrus, 11% Superior Frontal Gyrus
						HOSSA:<br>66% Right Cerebral Cortex, 30% Right Cerebral White Matter
6a	30.0	9.0	64.0	-4.81		HOCSA:<br>22% Middle Frontal Gyrus, 18% Superior Frontal Gyrus
						HOSSA:<br>62% Right Cerebral Cortex, 2% Right Cerebral White Matter
7	50.0	37.0	26.0	-5.31	6	HOCSA:<br>41% Frontal Pole, 16% Middle Frontal Gyrus
						HOSSA:<br>67% Right Cerebral Cortex, 1% Right Cerebral White Matter
						JHA:<br>12% GM Broca's area BA45 R
8	53.0	34.0	26.0	-4.88	2	HOCSA:<br>15% Middle Frontal Gyrus, 10% Frontal Pole
						HOSSA:

						38% Right Cerebral Cortex, 1% Right Cerebral White Matter
						JHA:<br>4% GM Broca's area BA45 R
9	-42.0	9.0	24.0	-4.77	344	HOCSA:<br>31% Inferior Frontal Gyrus, pars opercularis, 12% Precentral Gyrus, 5% Middle Frontal Gyrus
						HOSSA:<br>56% Left Cerebral Cortex, 44% Left Cerebral White Matter
						JHA:<br>32% GM Broca's area BA44 L
9a	-44.0	-3.0	42.0	-4.2		HOCSA:<br>35% Precentral Gyrus, 7% Middle Frontal Gyrus
						HOSSA:<br>51% Left Cerebral Cortex, 49% Left Cerebral White Matter
						JHA:<br>19% GM Premotor cortex BA6 L, 10% WM Corticospinal tract L, 3% GM Primary motor cortex BA4a L
9b	-34.0	-1.0	49.0	-4.16		HOCSA:<br>28% Precentral Gyrus, 17% Middle Frontal Gyrus, 1% Superior Frontal Gyrus
						HOSSA:<br>57% Left Cerebral Cortex, 43% Left Cerebral White Matter
						JHA:<br>18% GM Premotor cortex BA6 L, 8% GM Primary motor cortex BA4a L, 4% WM Corticospinal tract L
9c	-47.0	-1.0	52.0	-4.11		HOCSA:<br>52% Precentral Gyrus, 18% Middle Frontal Gyrus
						HOSSA:<br>81% Left Cerebral Cortex, 15% Left Cerebral White Matter
						JHA:

						81% GM Premotor cortex BA6 L, 1% GM Primary motor cortex BA4a L
10	-22.0	-26.0	24.0	-4.31	47	HOSSA:<br>95% Left Cerebral White Matter, 4% Left Lateral Ventricle, 1% Left Caudate
						JHA:<br>28% WM Corticospinal tract L, 19% WM Superior occipito-frontal fascicle L
10a	-9.0	-23.0	26.0	-3.88		HOSSA:<br>69% Left Cerebral White Matter, 31% Left Lateral Ventricle, 0% Left Cerebral Cortex
						JHA:<br>49% WM Callosal body, 2% WM Cingulum L

References

1. Daw, N. D., Gershman, S. J., Seymour, B., Dayan, P. & Dolan, R. J. Model-Based Influences on Humans' Choices and Striatal Prediction Errors. *Neuron* **69**, 1204–1215 (2011).
2. Constantinescu, A. O., O'Reilly, J. X. & Behrens, T. E. J. Organizing conceptual knowledge in humans with a gridlike code. *Science* **352**, 1464–1468 (2016).
3. Brady, T. F., Konkle, T., Alvarez, G. A. & Oliva, A. Visual long-term memory has a massive storage capacity for object details. *Proc. Natl. Acad. Sci.* **105**, 14325–14329 (2008).
4. Cichy, R. M., Pantazis, D. & Oliva, A. Similarity-Based Fusion of MEG and fMRI Reveals Spatio-Temporal Dynamics in Human Cortex During Visual Object Recognition. *Cereb. Cortex* **26**, 3563–3579 (2016).
5. Kiani, R., Esteky, H., Mirpour, K. & Tanaka, K. Object Category Structure in Response Patterns of Neuronal Population in Monkey Inferior Temporal Cortex. *J. Neurophysiol.* **97**, 4296–4309 (2007).
6. Konkle, T., Brady, T. F., Alvarez, G. A. & Oliva, A. Scene Memory Is More Detailed Than You Think: The Role of Categories in Visual Long-Term Memory. *Psychol. Sci.* **21**, 1551–1556 (2010).
7. Kriegeskorte, N. *et al.* Matching Categorical Object Representations in Inferior Temporal Cortex of Man and Monkey. *Neuron* **60**, 1126–1141 (2008).
8. Righi, G., Peissig, J. J. & Tarr, M. J. Recognizing disguised faces. *Vis. Cogn.* **20**, 143–169 (2012).
