# Supplementary material for: Grid-like entorhinal representation of an abstract value space during prospective decision making
Source: Nat Commun. 2024 Feb 9;15:1198. doi: 10.1038/s41467-024-45127-z (PMC10858181; doi:10.1038/s41467-024-45127-z)
Supplement: Supplementary file 3 — Reporting Summary [file 41467_2024_45127_MOESM3_ESM.pdf]

nature portfolio

Corresponding author(s): Alexander Nitsch

Last updated by author(s): Dec 6, 2023

Reporting Summary

Nature Portfolio wishes to improve the reproducibility of the work that we publish. This form provides structure for consistency and transparency in reporting. For further information on Nature Portfolio policies, see our Editorial Policies and the Editorial Policy Checklist.

Statistics

For all statistical analyses, confirm that the following items are present in the figure legend, table legend, main text, or Methods section.

n/a	Confirmed	
☐	☒	The exact sample size (*n*) for each experimental group/condition, given as a discrete number and unit of measurement
☐	☒	A statement on whether measurements were taken from distinct samples or whether the same sample was measured repeatedly
☐	☒	The statistical test(s) used AND whether they are one- or two-sided<br>*Only common tests should be described solely by name; describe more complex techniques in the Methods section.*
☐	☒	A description of all covariates tested
☐	☒	A description of any assumptions or corrections, such as tests of normality and adjustment for multiple comparisons
☐	☒	A full description of the statistical parameters including central tendency (e.g. means) or other basic estimates (e.g. regression coefficient) AND variation (e.g. standard deviation) or associated estimates of uncertainty (e.g. confidence intervals)
☐	☒	For null hypothesis testing, the test statistic (e.g. *F*, *t*, *r*) with confidence intervals, effect sizes, degrees of freedom and *P* value noted<br>*Give P values as exact values whenever suitable.*
☒	☐	For Bayesian analysis, information on the choice of priors and Markov chain Monte Carlo settings
☐	☒	For hierarchical and complex designs, identification of the appropriate level for tests and full reporting of outcomes
☐	☒	Estimates of effect sizes (e.g. Cohen's *d*, Pearson's *r*), indicating how they were calculated

Our web collection on statistics for biologists contains articles on many of the points above.

Software and code

Policy information about availability of computer code

Data collection	The prospective decision making task, the picture viewing task and the Santa Barbara Sense of Direction Scale (SBSOD) questionnaire (Hegarty, 2002) were programmed in Python 3.7 using the PsychoPy package (Peirce et al. (2019); version 3.1.5; https://lindeloev.net/psychopy-course/) in Spyder (https://www.spyder-ide.org/; version 4.0.0b3) distributed via Anaconda (https://www.anaconda.com/; version 2019.03). The instruction for the prospective decision making task was programmed using the Psycho Builder (Peirce et al. (2019); version 2020.2.3). For the two-stage task (Daw et al., 2011), a PsychoPy-based Python script from a publicly available repository (Abraham Nunes, https://abrahamnunes.github.io/paradigms/) was used. The script was adapted in Python 3.7 using the PsychoPy package (Peirce et al. (2019); version 3.1.5) in Spyder (https://www.spyder-ide.org/; version 4.0.0b3) distributed via Anaconda (https://www.anaconda.com/; version 2019.03).<br>MRI data were recorded using a 3 Tesla Siemens Magnetom Prisma Fit scanner (Siemens, Erlangen, Germany) with a 32-channel head coil. Stimuli for the fMRI tasks (prospective decision making task, picture viewing task) were projected on a screen via a mirror attached to the head coil and behavioral responses were collected with an MRI-compatible button box.

Data analysis	Behavioral and MRI analyses were carried out in Python 3.8 using Spyder (https://www.spyder-ide.org/; version 5.1.5) distributed via Anaconda (https://www.anaconda.com/; version 2020.11).<br>MRI analyses were carried out using the tools dcm2bids (version 2.1.6, https://unfmontreal.github.io/Dcm2Bids/), pydeface (version 2.0.0, https://github.com/poldracklab/pydeface), fMRIPrep 20.2.6 (Esteban et al., 2018; Esteban et al., 2022; RRID:SCR_016216), Python packages nilearn (version 0.9.0), nibabel (version 3.2.1), scikit-learn (version 1.0.1) as well as FSL (version 6.0.3) and ANTS (version 2.3.5).<br>Statistical analyses were based on the Python packages scipy (version 1.10.0) and statsmodels (version 0.13.2).<br><br>Analysis code is available on Github (https://github.com/nitschalex/Paper_Value_Space).

For manuscripts utilizing custom algorithms or software that are central to the research but not yet described in published literature, software must be made available to editors and reviewers. We strongly encourage code deposition in a community repository (e.g. GitHub). See the Nature Portfolio guidelines for submitting code & software for further information.

Data

Policy information about availability of data

All manuscripts must include a data availability statement. This statement should provide the following information, where applicable:

- Accession codes, unique identifiers, or web links for publicly available datasets
- A description of any restrictions on data availability
- For clinical datasets or third party data, please ensure that the statement adheres to our policy

Data to reproduce the statistical analyses reported in this paper are available on the Open Science Framework (https://osf.io/z4k5v/). Source data are provided with this paper.
Task stimuli are available in public stimulus datasets (Brady et al., 2008; Cichy et al., 2016; Kiani et al., 2007; Konkle et al., 2010; Kriegeskorte et al., 2008; Righi et al., 2012). The Harvard-Oxford Cortical and Subcortical Structural Atlases and the Juelich Histological Atlas used for the neuroimaging analyses are provided by FSL.

Research involving human participants, their data, or biological material

Policy information about studies with human participants or human data. See also policy information about sex, gender (identity/presentation), and sexual orientation and race, ethnicity and racism.

Reporting on sex and gender	Gender of participants was self-reported and was not considered in the analyses.
Reporting on race, ethnicity, or other socially relevant groupings	No socially relevant groupings were considered in the study.
Population characteristics	51 participants took part in the study. All participants had normal or corrected-to-normal vision, no history of or current neurological or psychiatric disorders, were right-handed and met MRI safety criteria. The final sample used for the analyses consisted of 46 participants (age: M = 28.15 years, SD = 4.77 years, range = 19-39 years; 25 female).
Recruitment	Participants were recruited using the participant database of the Max Planck Institute for Human Cognitive and Brain Sciences, Leipzig, Germany. Participants tended to be undergraduate or Master's students at the University of Leipzig and reflected the demographics of the local student population. We are not aware of any selection biases (self or others) that could have impacted the results.
Ethics oversight	The study was approved by the ethics committee at the Medical Faculty at the University of Leipzig (421/19-ek).

Note that full information on the approval of the study protocol must also be provided in the manuscript.

Field-specific reporting

Please select the one below that is the best fit for your research. If you are not sure, read the appropriate sections before making your selection.

☒ Life sciences ☐ Behavioural & social sciences ☐ Ecological, evolutionary & environmental sciences

For a reference copy of the document with all sections, see nature.com/documents/nr-reporting-summary-flat.pdf

Life sciences study design

All studies must disclose on these points even when the disclosure is negative.

Sample size	51 participants took part in the study. The sample size was determined by a power analysis using G*Power (Faul et al., 2007). This yielded a necessary sample size of 41 participants to achieve a statistical power of 80 % for a small-to-medium effect size (d = 0.4, α = 0.05, one-tailed t-test). Additionally, 10 participants were recruited to account for potential dropouts.
Data exclusions	For the data analysis, one participant was excluded due to missing fMRI data because of technical problems during data acquisition. Furthermore, four participants were excluded due to low performance of the prospective decision making task (performance criterion of 70% accuracy based on previous piloting).

Replication	One fMRI study was conducted and group-level replication was not undertaken because of the resources needed for running this fMRI study. The main effects are present in the majority of participants.
Randomization	Only one group of participants was tested and thus participants were not assigned to experimental groups.
Blinding	No group assignment took place, blinding was therefore not necessary.

Reporting for specific materials, systems and methods

We require information from authors about some types of materials, experimental systems and methods used in many studies. Here, indicate whether each material, system or method listed is relevant to your study. If you are not sure if a list item applies to your research, read the appropriate section before selecting a response.

Materials & experimental systems

n/a	Involved in the study
☒	☐ Antibodies
☒	☐ Eukaryotic cell lines
☒	☐ Palaeontology and archaeology
☒	☐ Animals and other organisms
☒	☐ Clinical data
☒	☐ Dual use research of concern
☒	☐ Plants

Methods

n/a	Involved in the study
☒	☐ ChIP-seq
☒	☐ Flow cytometry
☐	☒ MRI-based neuroimaging

Magnetic resonance imaging

Experimental design

Design type	Task fMRI with event-related design
Design specifications	The prospective decision making task consisted of four blocks (fMRI runs), with 36 trials per block (total: 144 trials). A trial consisted of multiple events (referred to as time points) reflecting value changes over time, with each time point being presented for 2.5 s. Active trials involved an observation phase of 3-5 time points (7.5 - 12.5 s) and an active choice (self-paced, max. 3 s), followed by an inter-stimulus interval sampled from a truncated exponential distribution (min = 3 s, max = 8 s, mu = 4 s, sampled mean = 4.1 s) and feedback for 2.5 s. Passive trials involved 6 time points (15s). Inter-trial intervals were sampled from a truncated exponential distribution (min = 3 s, max = 8 s, mu = 4 s, sampled mean = 4.1 s). Each task block lasted approx. 13 min (M = 12.74 min, SD = 0.15 min).<br><br>The picture viewing task consisted of 65 trials. In a trial, a stimulus was presented for 2 s, followed by an inter-trial interval sampled from a truncated exponential distribution (min = 2 s, max = 8 s, mu = 3 s, sampled mean = 3.3 s). The task lasted approx. 6 min (M = 6.18 min, SD = 0.02 min).
Behavioral performance measures	In the scanner, behavioral responses (button presses, response times) were collected with an MRI-compatible button box. We assessed performance as the proportion of correctly answered trials in both tasks, with a performance criterion of at least 70 % in the prospective decision making task to be included in the final analysis sample.

Acquisition

Imaging type(s)	functional and structural MRI , fieldmap
Field strength	3T
Sequence & imaging parameters	MRI data were recorded using a 3 Tesla Siemens Magnetom Prisma Fit scanner (Siemens, Erlangen, Germany) with a 32-channel head coil.<br>After a localizer scan, functional scans (fMRI) for the picture viewing task and the four runs of the prospective decision making task were acquired using T2*-weighted whole-brain gradient-echo echo planar imaging (GE-EPI) with multiband acceleration, sensitive to blood-oxygen-level-dependent (BOLD) contrast (Feinberg et al., 2010; Moeller et al., 2010). Settings of the fMRI sequence were as follows: TR = 1500 ms; TE = 22 ms; voxel size = 2.5 mm isotropic; field of view = 204 mm; flip angle = 70°; partial fourier = 0.75; bandwidth = 1794 Hz/Px; multi-band acceleration factor = 3; 69 slices interleaved; distance factor = 0 %; phase encoding direction = A-P. On average, 253 volumes were recorded for the PVT (M = 252.76 volumes, SD = 5.45 volumes) and 514 volumes per run of the prospective decision making task (M = 513.70 volumes, SD = 7.57 volumes).<br>After the second run of the prospective decision making task, field maps were acquired to measure and later correct for magnetic field inhomogeneities. Field maps were acquired using both opposite phase-encoded EPIs and a double echo sequence. Settings of the opposite phase-encoded EPIs were as follows: TR = 8000 ms; TE = 50 ms; voxel size = 2.5 mm isotropic; field of view = 204 mm; flip angle = 90°; partial fourier = 0.75; bandwidth = 1794 Hz/Px; multi-band acceleration factor = 1; 69 slices interleaved; distance factor = 0 %. Settings of the double echo sequence were as follows: TR = 620 ms; TE1 = 4.00 ms; TE2 = 6.46 ms; voxel size = 2.5 mm isotropic; field of view = 204 mm; flip angle =

60°; bandwidth = 412 Hz/Px; 69 slices interleaved; distance factor = 0 %.
At the end of the scanning session, a T1-weighted MPRAGE anatomical scan was acquired (TR = 2300 ms; TE = 2.98 ms; voxel size = 1 mm isotropic; field of view = 256 mm; flip angle = 9°; bandwidth = 240 Hz/Px; distance factor = 50 %).

Area of acquisition: whole-brain

Diffusion MRI ☐ Used ☒ Not used

Preprocessing

Preprocessing software

We converted DICOM files of the MRI scanner to NIfTI files and reorganized them according to the BIDS standard (K. J. Gorgolewski et al., 2016) using the tool dcm2bids (version 2.1.6, https://unfmontreal.github.io/Dcm2Bids/). Furthermore, we removed facial structure in the anatomical scan using the tool pydeface (version 2.0.0, https://github.com/poldracklab/pydeface) to further anonymize the data. Further preprocessing was performed using fMRIPrep 20.2.6 (Esteban et al., 2018; Esteban et al., 2022; RRID:SCR_016216), which is based on Nipype 1.7.0 (Esteban et al., 2022; Gorgolewski et al., 2011; RRID:SCR_002502).
For univariate analyses, the data were spatially smoothed with a 6 mm full-width at half maximum Gaussian filter (FWHM) using nilearn (version 0.9.0, within the FirstLevelModel class). For the multivariate choice decoding analysis, no smoothing was applied to preserve differences between voxels.

Normalization

In the fMRIPrep pipeline, the BOLD time-series were resampled onto their original, native space by applying a single, composite transform to correct for head-motion and susceptibility distortions. Furthermore, the BOLD time-series were resampled into MNI152NLin2009cAsym standard space (see fMRIPrep details below).
The T1-weighted (T1w) image was corrected for intensity non-uniformity (INU) with N4BiasFieldCorrection (Tustison et al., 2010), distributed with ANTs 2.3.3 (Avants et al., 2008, RRID:SCR_004757), and used as T1w-reference throughout the workflow. The T1w-reference was then skull-stripped with a Nipype implementation of the antsBrainExtraction.sh workflow (from ANTs), using OASIS30ANTs as target template. Brain tissue segmentation of cerebrospinal fluid (CSF), white-matter (WM) and gray-matter (GM) was performed on the brain-extracted T1w using fast (FSL 5.0.9, RRID:SCR_002823, Zhang et al., 2001). Brain surfaces were reconstructed using recon-all (FreeSurfer 6.0.1, RRID:SCR_001847, Dale et al., 1999), and the brain mask estimated previously was refined with a custom variation of the method to reconcile ANTs-derived and FreeSurfer-derived segmentations of the cortical gray-matter of Mindboggle (RRID:SCR_002438, Klein et al., 2017). Volume-based spatial normalization to two standard spaces (MNI152NLin2009cAsym, MNI152NLin6Asym) was performed through nonlinear registration with antsRegistration (ANTs 2.3.3), using brain-extracted versions of both T1w reference and the T1w template. The following templates were selected for spatial normalization: ICBM 152 Nonlinear Asymmetrical template version 2009c [Fonov et al., 2009, RRID:SCR_008796; TemplateFlow ID: MNI152NLin2009cAsym], FSL's MNI ICBM 152 non-linear 6th Generation Asymmetric Average Brain Stereotaxic Registration Model [Evans et al., 2012, RRID:SCR_002823; TemplateFlow ID: MNI152NLin6Asym].

Normalization template

ICBM 152 Nonlinear Asymmetrical template version 2009c [Fonov et al., 2009, RRID:SCR_008796; TemplateFlow ID: MNI152NLin2009cAsym]

Noise and artifact removal

To control for noise signals in the fMRI data, the first-level GLMs included 37 confound regressors estimated during preprocessing with fMRIPrep. Following the denoising strategy proposed by Satterthwaite et al. (2013), these confounds included 24 motion parameters (6 basic translation / rotation parameters, 6 temporal derivatives of these and 12 quadratic terms of the basic parameters and their derivatives) as well as 12 global signal parameters (3 basic average CSF, WM and global signal parameters, 3 temporal derivatives of these and 6 quadratic terms of the basic parameters and their derivatives). Additionally, the confounds included framewise displacement as a summary metric of frame-to-frame head motion. Furthermore, the GLMs included discrete cosine-basis regressors estimated by fMRIPrep to account for temporal low-frequency signal drifts.

Volume censoring

No volume censoring was performed.

Statistical modeling & inference

Model type and settings

We used both univariate (hexadirectional signals, value difference analysis) and multivariate (choice decoding) analyses. In each analysis, we first estimated effects / contrasts for each subject as a first-level fixed-effects analysis and then used second-level group inferential statistics.

Second-level: For univariate analyses, we tested significance across participants using non-parametric permutation testing implemented in FSL Randomise with 10000 permutations. We used threshold-free cluster enhancement and corrected for multiple comparisons with family-wise error rate (pFWE < 0.05). For the multivariate choice decoding ROI analysis, we tested participant-specific z-scores against 0 using one-sample t-tests.

Effect(s) tested

Brief description of analyses (see Methods for details)

Analysis of hexadirectional signals (grid-like representation)
The analysis consists of two steps: In the first step, the grid orientation is estimated and in the second step the prediction of hexadirectional modulation according to the grid orientation is tested using independent data. We implemented a cross-validation procedure, estimating the putative entorhinal grid orientation using three of four task runs and testing for a hexadirectional modulation aligned to the orientation in the left-out test run (based on Doeller et al., 2010; Nau et al., 2018). In the estimation set (three of four runs, GLM1), the GLM included trajectory regressors which were modulated by the sine and cosine of the direction (angle) θ of the trajectory with 60° (6-fold) periodicity (sin(6*θt) and cos(6*θt)). Effect sizes of these regressors were used to estimate the grid orientation in entorhinal cortex. In the independent test set (left-out run,

GLM2), the GLM included a parametrically modulated trajectory regressor reflecting a six-fold (hexadirectional) sinusoidal modulation based on the mean entorhinal grid orientation (cos(6*(θt - Θ))). Effect sizes of the parametric cosine regressor were averaged across the four cross-validation folds (fixed effects) to obtain an overall effect size.

Value difference analysis
The GLM included regressors for choice time points which were parametrically modulated regressors by the values of the chosen and unchosen option, respectively. We then contrasted the effect sizes of the chosen value vs. the unchosen value regressor [1, -1] to test for a modulation of activity by the value difference.

Choice decoding analysis
We first estimated neural activation patterns of trials in the picture viewing task (PVT, training data) and of choice time points in the prospective decision making task (test data) using GLMs. We used z-scores of the trial and choice regressors for the next steps. We z-standardized the data run-wise. We then trained a decoder to distinguish neural activation patterns of the four category-specific stimuli based on the PVT data (support vector classifier, regularization parameter C = 1.0, kernel = rbf, probability = True to enable probability estimates). Subsequently, we applied this decoder to the neural activation patterns of choices in the prospective decision making task. More specifically, we extracted the probabilities which the decoder assigned to each of the four stimuli and computed two difference scores for each choice: 1. on-screen high-value vs. low-value stimulus, 2. off-screen congruent high-value vs. low-value stimulus. To compare these difference scores against chance level performance of the decoder, we implemented a permutation test, repeating this procedure 1000 times with randomly permuted trial labels in the PVT training data. For each choice, we then converted the original difference scores to z-scores based on the null distribution generated by the permutations. Lastly, we averaged z-scores across choices to obtain two summary scores per participant.

Specify type of analysis: ☐ Whole brain ☐ ROI-based ☒ Both

Anatomical location(s)

For our hypothesis of a grid-like representation in the entorhinal cortex, we used participant-specific bilateral entorhinal cortex masks created by FreeSurfer segmentations of the participants' anatomical images during preprocessing with fMRIPrep (FreeSurfer labels 1006 & 2006). For small volume correction on the group level, we created an entorhinal cortex mask based on both participant-specific anatomy (Freesurfer masks) and MNI standard atlas labeling (entorhinal cortex mask of the Juelich Histological Atlas provided by FSL, thresholded at 50% probability). To further explore vmPFC representations, we defined two ROIs as spheres with a 7 mm radius 1) around the peak voxel of our value difference analysis in vmPFC (MNI peak voxel coordinates: 3,42,-8; 89 voxels) and 2) around the peak voxel of the hexadirectional effect reported by Constantinescu et al. (2016) in vmPFC (MNI peak voxel coordinates: 16,54,-2; 95 voxels).

For our choice decoding hypothesis, we leveraged neural responses to category-specific stimuli (faces, tools, scenes, body parts) in category-selective regions of the occipital-temporal cortex. We created participant-specific occipital-temporal ROI masks as follows. First, we thresholded occipital and temporal lobe probability masks of the MNI Structural Atlas provided by FSL (version 6.0.3) at a threshold of 25 % and created their union. We then transformed this MNI-based mask to each participants' native space using ANTS (version 2.3.5) and resampled it to the resolution of the functional data based on transformation files created during preprocessing with fMRIPrep. We intersected these with participant-specific gray matter masks. For this purpose, we thresholded gray matter probability masks created by fMRIPrep's segmentation of the anatomical image at a threshold of 50 % and resampled them to the functional resolution. In the decoding analysis, we used these participant-specific gray matter occipital-temporal masks for additional feature selection based on univariate stimulus-category effects in the PVT training data (ANOVA F-values between each feature/voxel and the trial labels, 20 % of the voxels with the highest F-values selected) to create the final masks.

Statistic type for inference

(See Eklund et al. 2016)

Whole-brain analyses: threshold-free cluster enhancement
ROI analyses: mean effect averaged over voxels of the ROI, permutation-based significance test

Correction

FWE based on FSL Randomise (with small volume correction in the entorhinal cortex for hexadirectional analysis and whole-brain)

Models & analysis

n/a	Involved in the study
☒	☐ Functional and/or effective connectivity
☒	☐ Graph analysis
☐	☒ Multivariate modeling or predictive analysis

Multivariate modeling and predictive analysis

see above: Effects tested and anatomical location for choice decoding analysis
